# Chondroitin sulfate reinforces mitochondrial redox homeostasis to enable integrated intervertebral disc regeneration

**DOI:** 10.1093/rb/rbag134

**Published:** 2026-06-15

**Authors:** Xin Wang, Yixue Huang, Yilin Yang, Yesheng Jin, Yun Xiao, Hao Liu, Yong Xu, Yijie Liu

**Affiliations:** Department of Orthopaedic Surgery, The Fourth Affiliated Hospital of Soochow University, Suzhou Medical College, Soochow University, Suzhou 215000, China; Department of Orthopaedics, The First Affiliated Hospital of Soochow University, Orthopaedic Institute, Suzhou Medical College, Soochow University, Suzhou 215006, China; Department of Orthopaedics, The First Affiliated Hospital of Soochow University, Orthopaedic Institute, Suzhou Medical College, Soochow University, Suzhou 215006, China; Department of Orthopaedics, The First Affiliated Hospital of Soochow University, Orthopaedic Institute, Suzhou Medical College, Soochow University, Suzhou 215006, China; Department of Orthopaedics, The First Affiliated Hospital of Soochow University, Orthopaedic Institute, Suzhou Medical College, Soochow University, Suzhou 215006, China; Department of Orthopaedics, The First Affiliated Hospital of Soochow University, Orthopaedic Institute, Suzhou Medical College, Soochow University, Suzhou 215006, China; Department of Orthopaedics, The First Affiliated Hospital of Soochow University, Orthopaedic Institute, Suzhou Medical College, Soochow University, Suzhou 215006, China; National Center for Translational Medicine (Shanghai) SHU Branch, Shanghai University, Shanghai 200444, China; Department of Orthopaedic Surgery, The Fourth Affiliated Hospital of Soochow University, Suzhou Medical College, Soochow University, Suzhou 215000, China

**Keywords:** intervertebral disc degeneration, chondroitin sulfate, oxidative stress, mitochondrial function, PI3K-Akt pathway

## Abstract

Intervertebral disc degeneration (IVDD), characterized by oxidative stress, mitochondrial dysfunction and extracellular matrix (ECM) breakdown, is a major contributor to low back pain. Current regenerative strategies often fail to address both the biological and structural aspects of IVDD in an integrated manner. Here, we demonstrate that chondroitin sulfate (CS) exerts a dual therapeutic role by enhancing endogenous antioxidant defenses and promoting anabolic ECM synthesis in both annulus fibrosus cells (AFCs) and nucleus pulposus cells (NPCs). Mechanistically, CS activates the integrin α4β1-PI3K-Akt signaling pathway, preserving mitochondrial integrity and restoring redox homeostasis. Furthermore, we developed an injectable, multifunctional PVA/collagen (Col)/CS hydrogel that integrates mechanical support, bioactivity and targeted CS delivery to enable unified repair of the disc’s biphasic structure. In a rat IVDD model, this hydrogel effectively preserved disc architecture, restored disc height and MRI signal and upregulated key matrix and antioxidant markers, demonstrating coordinated repair of both the annulus fibrosus (AF) and the nucleus pulposus (NP). Our study establishes CS as a potent dual-function agent and presents an integrated hydrogel-based strategy for functional disc regeneration.

## Graphical abstract

**Graphical Abstract:**
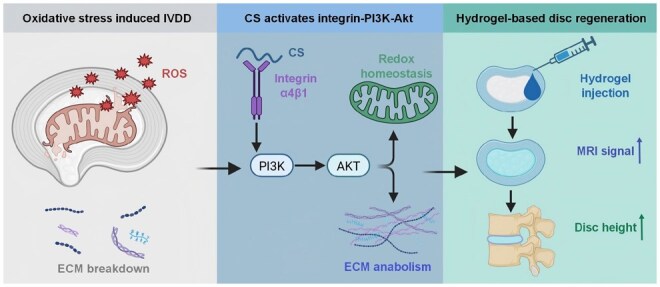

## Introduction

As a primary contributor to low back pain, intervertebral disc degeneration (IVDD) represents a substantial worldwide health burden, resulting in disability-adjusted life years and imposing substantial healthcare and societal costs [1, 2]. Cellular senescence initiates a deleterious cascade in which the resulting extracellular matrix (ECM) anabolic–catabolic imbalance, when combined with sustained mechanical overloading, propels a vicious circle between oxidative stress and inflammation [3–6]. Oxidative damage and inflammation not only accelerate catastrophic tissue breakdown but also facilitate aberrant innervation and angiogenesis, ultimately manifesting as the debilitating pain and disability characteristic of advanced degeneration [7–9]. Current pain management or invasive spinal fusion is primarily palliative. However, current regenerative paradigms, which encompass techniques utilizing biomaterials, stem cell-based interventions, genetic modulation and engineered tissue methods, frequently aim to rehabilitate either the nucleus pulposus (NP) or the annulus fibrosus (AF) in isolation. Recently, smart materials that are capable of responding to endogenous (e.g. pH, enzymes) or exogenous (e.g. light, ultrasound, magnetic field) stimuli have emerged as a promising platform for disc tissue engineering [10]. Despite these advances, most existing strategies still target only the NP or the AF individually. This singular focus fails to achieve integrated repair and the simultaneous restoration of adequate biomechanical performance [11]. Consequently, an integrated strategy aimed at achieving endogenous regeneration and metabolic rebalancing of disc cells is emerging as a new paradigm for treating IVDD.

**Figure 1:**
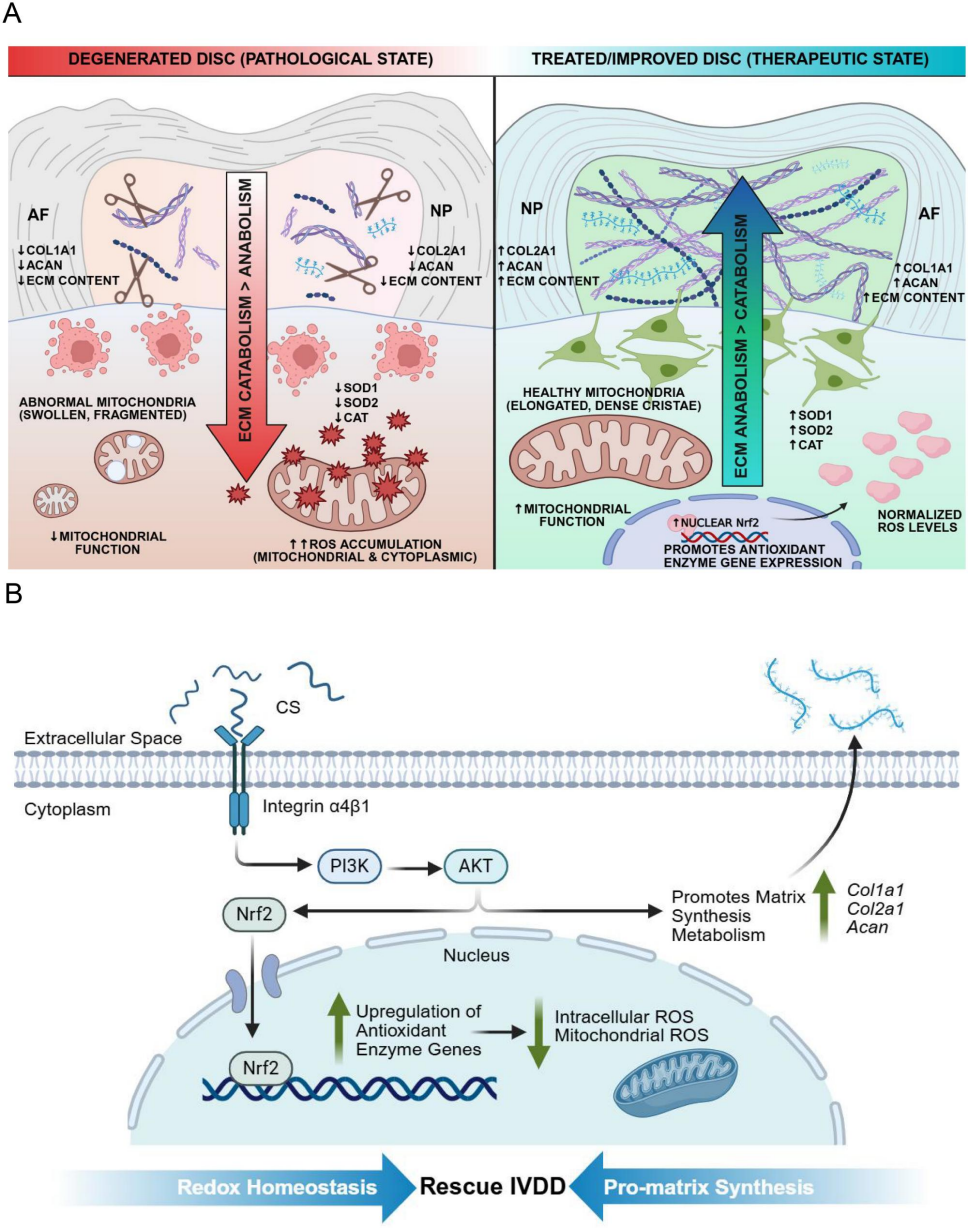
Proposed mechanism by which CS alleviates IVDD. (**A**) In contrast to the degenerative state characterized by suppressed matrix synthesis and enhanced catabolism, CS intervention rescues disc homeostasis by upregulating the endogenous antioxidant enzyme system, improving mitochondrial function and thereby promoting matrix synthesis in both nucleus pulposus and AFCs. (**B**) Mechanistically, CS activates the integrin α4β1-PI3K-AKT signaling axis, which enhances antioxidant capacity, reduces intracellular ROS, upregulates anabolic gene expression and ultimately attenuates IVDD progression.

The avascular nucleus pulposus inherently exists in a hypoxic milieu. Under pathological conditions such as endplate calcification and inflammation, this environment transitions from a physiological to a severely pathological hypoxia, triggering a deleterious cascade of mitochondrial dysfunction and rampant reactive oxygen species (ROS) generation [12, 13]. ROS orchestrate a cascade of destruction in nucleus pulposus cells (NPCs) by directly damaging cellular constituents (DNA, proteins and lipids), compromising matrix synthesis, instigating a potent inflammatory cascade and consequently upregulating catabolic enzymes (MMPs and ADAMTS), ultimately driving the irreversible breakdown of the ECM (Figure 1A) [14, 15]. There is growing consensus on the critical involvement of oxidative stress in IVDD [16, 17]. This intricate phenomenon is under precise regulation within the mitochondrion. Paradoxically, while the mitochondrial electron transport chain (ETC) constitutes a major source of ROS, it also harbors a dedicated and efficient antioxidant enzyme system-comprising superoxide dismutase (SOD), glutathione peroxidase and catalase, which constitutes a specialized ROS scavenging system [18]. Compromised superoxide dismutase 2 (SOD2) function leads to a disruption of redox homeostasis, which in turn induces and exacerbates IVD degeneration [19]. Upregulation of GPX7 effectively rescued senescence in NPCs and mitigated IVDD in rats [20]. We hypothesize that augmenting the endogenous antioxidant enzyme system can scavenge aberrant ROS, thereby maintaining redox homeostasis and safeguarding energy supply to ultimately mitigate the progression of IVDD (Figure 1A).

The ECM comprises a dynamic assembly of fibrillar proteins, proteoglycans and glycosaminoglycans (GAG). It transcends its structural role by providing mechanical support and serving as a critical signaling hub that actively regulates cellular behavior and tissue homeostasis [21]. A major constituent of this ECM is chondroitin sulfate (CS), which belongs to the sulfated GAG family and is renowned for its high negative charge and exceptional hydrophilicity. The spatiotemporally specific patterns of CS sulfation orchestrate pivotal events in the ontogeny of the rat IVD [22]. Moreover, a differential reduction in total CS and its specific subtypes (CS-A and CS-C) has been observed in degenerated intervertebral discs [23]. Beyond its fundamental structural role, CS exhibits a spectrum of potent bioactivities, including anti-inflammatory, antioxidant, anti-thrombotic and immunomodulatory effects [24]. However, while the anti-inflammatory and matrix-anabolic functions of CS are well-established in the context of osteoarthritis [25–27], its potential therapeutic role in IVDD remains to be elucidated. Herein, we demonstrate that CS potently enhances the expression of endogenous antioxidant enzymes in disc cells, effectively clearing excess ROS in the degenerative microenvironment. This restoration of redox homeostasis and cellular bioenergetics subsequently rescues the matrix synthesis capacity of both annulus fibrosus cells (AFCs) and NPCs (Figure 1A). Mechanistically, we delineate that CS orchestrates its protection through the activation of the PI3K-Akt axis, which in turn safeguards mitochondrial integrity and fosters an anabolic cellular phenotype (Figure 1B).

Injectable hydrogels have gained prominence as an attractive therapeutic approach for intervertebral disc regeneration, offering a potential means to restore disc height and deliver therapeutic agents *in situ* [28–31]. However, current hydrogel designs often fall short of addressing the disc’s complex biphasic structure and multifactorial pathophysiology, typically focusing on either the NP or the AF in isolation [32, 33]. To overcome these limitations, we engineered a multifunctional composite hydrogel comprising three key components: a mechanically adaptive polyvinyl alcohol (PVA) network for dynamic load-bearing support, a collagen framework to mimic the native ECM, and CS for targeted therapeutic delivery. This integrated system is designed to simultaneously mitigate the oxidative stress microenvironment, promote ECM synthesis and restore the disc’s structural and functional integrity.

## Materials and methods

### Human IVD tissue samples

Ethical approval for human intervertebral disc samples was obtained from the Institutional Review Board at the Fourth Affiliated Hospital of Soochow University (application number 2025-251345). Written consent was obtained from all parties concerning the use of surgical discard tissues (Supplementary Table S1). Specimens were processed by fixation, paraffin embedding and sectioning. Histological evaluations, including Hematoxylin and Eosin (H&E), Alcian Blue staining and Immunohistochemistry (IHC) for CS (Sigma-Aldrich, SAB4200696), were performed on these sections. The degenerative state of each disc was classified based on preoperative magnetic resonance imaging (MRI) scans as defined by Pfirrmann grades.

### Isolation and culture of rat caudal intervertebral disc cells

NP and AF tissues were aseptically harvested from the caudal IVDs of 6-week-old Sprague-Dawley rats (200 ± 20 g). The isolated tissues were minced and underwent sequential enzymatic digestion using collagenase type I and II solutions, separately (0.2%) (OriLeaf, China, S10053, S10054). Following isolation, cells were cultured in complete DMEM/F12 medium (10% FBS, 1% pen/strep) with incubation at 37°C under 5% CO_2_ Experiments utilized cells between passages 1 and 4 cultured with 10% FBS.

### Gene expression analysis by qRT-PCR

The TRIzol method was employed to extract total RNA from cultured cells. Subsequent cDNA synthesis was carried out with a commercial reverse transcription kit. qRT-PCR amplification was performed on a CFX96 RealTime PCR system (Bio-RAD) with SYBR Green Master Mix. The expression levels of target genes were normalized to Gapdh and analyzed via the 2^(-ΔΔCt) method. All primer sequences are provided in Supplementary Table S2.

### Western blot

For protein extraction, cells were lysed on ice for 30 min using RIPA buffer containing 1% protease and phosphatase inhibitor cocktails. Following lysis, the homogenates were centrifuged at 13 000 rpm for 30 min to pellet cellular debris. The protein concentration in the resulting supernatant was then quantified using a BCA assay kit (Beyotime, China) according to the manufacturer’s instructions. Equal amounts of protein from each sample were separated by SDS-PAGE. The separated proteins were subsequently electrophoretically transferred onto PVDF membranes. To prevent non-specific binding, the membranes were blocked with 5% BSA for 1 h at room temperature. For immunoblotting, the blocked membranes were incubated with specific primary antibodies diluted in blocking buffer at 4°C for 12 h. The primary antibody panel comprised: anti-COL1A1 (Abclonal, A22090), anti-COL2A1 (Abcam, ab188570), anti-ACAN (Abclonal, A11691), anti-NRF2 (CST, 33649), anti-p-AKT (Proteintech, 66444-1-lg), anti-SOD1 (Abcam, ab51254), anti-SOD2 (Abcam, ab137037), anti-AKT (Proteintech, 10176-2-AP), anti-p-PI3K (Abclonal, AP0427), anti-PI3K (Proteintech, 60225-1-lg) and anti-β-actin (Abways, AB0035). The immunoblots were detected by enhanced chemiluminescence (ECL) after incubation with HRP-conjugated secondary antibodies (1–2 h). The resulting signals were captured, and the band intensities were analyzed via semi-quantitative densitometry using ImageJ. Uncropped raw Western blot images are provided in Supplementary Figure S8.

### Intracellular ROS detection by DCFH-DA staining

We assessed intracellular ROS levels with a commercial ROS assay kit (Beyotime, China, S0033S). Cells were exposed to ROS staining solution for 20–30 min. To identify nuclei, Hoechst 33342 (5 µg mL^−1^, Beyotime, China, C1011) was used as a counterstain. Fluorescence imaging was immediately conducted utilizing the ZEISS Axio Observer Z1 microscope (excitation/emission: 488 nm/525 nm for DCFH-DA; 350 nm/461 nm for Hoechst). ImageJ software was employed to quantify the DCFH-DA fluorescence intensity.

### JC-1 staining

Changes in mitochondrial membrane potential (ΔΨm) were evaluated using the JC-1 staining kit (Nanjing Jiancheng, China, G009-1-3) according to the manufacturer’s instructions. The shift from red fluorescence (J-aggregates, indicative of high ΔΨm) to green fluorescence (J-monomers, indicative of low ΔΨm) was captured by fluorescence microscopy (ZEISS, Axio Observer Z1). Quantification was achieved by measuring red and green fluorescence intensities separately and then computing their ratio.

### Mitochondrial network visualization with MitoTracker staining

To observe mitochondrial morphology, staining of cells was performed with MitoTracker Green (Beyotime, China, C1048) as per the provided protocol. To identify nuclei, Hoechst 33342 (5 µg mL^−1^, Beyotime, China, C1011) was used as a counterstain. Fluorescence microscopy was used to capture images of the mitochondrial network (excitation/emission: 490 nm/516 nm) and nuclei (350 nm/461 nm).

### Cell viability assessment by live/dead staining

The cytocompatibility of hydrogel extracts was determined using a Live/Dead viability/cytotoxicity assay. AFCs and NPCs were seeded in culture plates and treated with hydrogel extracts for 3 days. Staining of cells was performed with calcein-AM (labeling live cells) and propidium iodide (labeling dead cells) for 20 min at 37°C. A fluorescence microscope was used for imaging. The percentages of viable and non-viable cells were determined from three independent replicates.

### Cell proliferation assay

Proliferation of AFCs and NPCs was monitored with the Cell Counting Kit-8 (CCK-8, Beyotime, China, C0038). A seeding density of 10³ cells/well was used. After treatment with hydrogel extracts or CS subtypes, CCK-8 solution (10 µL) was introduced into every well at each time point and then incubated at 37°C. Absorbance at 450 nm was recorded with a microplate reader (BioTek, USA).

### Immunofluorescence staining

Cells grown on coverslips were fixed, permeabilized and blocked. They were incubated overnight at 4°C with primary antibodies targeting COL1A1 (Abclonal, A22090), COL2A1 (Abcam, ab34712) and SOD1 (Abcam, ab51254). After washing, appropriate fluorescent dye-conjugated secondary antibodies were applied. To identify nuclei, Hoechst 33342 (5 µg mL^−1^, Beyotime, China, C1011) was used as a counterstain. Microscopic observation and image acquisition were performed on an inverted fluorescence microscope (ZEISS, Axio Observer Z1).

### Senescence-associated **β**-galactosidase staining

SA-β-Gal activity, a marker of cellular senescence, was detected with a specific kit (Beyotime, C0602). Following fixation, the samples were subjected to incubation with the staining solution for 12 h at 37°C. Images of the blue-stained senescent cells were captured under a light microscope, and we quantified the proportion of positive cells.

### Transmission electron microscopy

Samples were initially fixed in 2.5% glutaraldehyde. The fixative was then removed, and the samples were rinsed three times (15 min each) with 0.1 M phosphate buffer (pH 7.0). This was followed by post-fixation in 1% osmium tetroxide for 1–2 h. After carefully discarding the osmium tetroxide, the samples were again washed three times with the same phosphate buffer (15 min per wash). Dehydration was carried out using a graded ethanol series (30%, 50%, 70%, 80%, 90% and 95%, 15 min each) followed by two changes of 100% ethanol (20 min each). The samples were then transitioned to pure acetone for 20 min. For infiltration, the samples were treated with a mixture of embedding resin and acetone (1:1 v/v) for 1 h, followed by a mixture with a higher resin ratio (3:1 v/v) for 3 h and finally with pure embedding resin overnight. The infiltrated samples were embedded in fresh resin and polymerized at 70°C overnight. Ultrathin sections (70–90 nm thick) were obtained using a LEICA EM UC7 ultramicrotome. The sections were doubly stained with uranyl acetate (saturated solution in 50% ethanol) and lead citrate, each for 5–10 min, air-dried and subsequently examined under a transmission electron microscope to assess mitochondrial ultrastructure.

### Measurement of MDA content and total SOD activity

The levels of malondialdehyde (MDA) and the enzymatic activity of total SOD in cell lysates were quantified using specific colorimetric assay kits (Nanjing Jiancheng, China, A003-1-2 for MDA, A001-3-1 for SOD), strictly adhering to the provided protocols. Absorbance readings were taken on a microplate reader.

### Transcriptome sequencing and bioinformatic analysis

RNA was purified from NPCs following treatment with or without CS. After quality control, sequencing libraries were constructed and sequenced on an Illumina platform. Differentially expressed genes (DEGs) were identified, and their biological functions were investigated through Gene Ontology (GO) and Kyoto Encyclopedia of Genes and Genomes (KEGG) pathway enrichment analyses using standard bioinformatics workflows.

### Characterization of PVA/Col/CS hydrogel

#### Synthesis of PVA/Col/CS hydrogel

Stock solutions were prepared by dissolving PVA (20% w/v), CS (1% w/v), type I collagen (10% w/v) and boric acid (10% w/v) separately in deionized water. The hydrogel precursor was formulated by mixing these solutions in a volume ratio of PVA: Collagen: CS: Boric acid = 2:1:1:1. The mixture was vortexed thoroughly and allowed to undergo crosslinking at room temperature to form a stable hydrogel.

#### Gelation, injectability and adhesiveness

The gelation ability was assessed by inverting the tube containing the mixed precursor solution. Injectability was evaluated by loading the pre-gel solution into a 23‑gauge syringe and extruding it through the needle. Adhesive property was tested by inverting a vial containing the fully crosslinked hydrogel onto a biological surface (a finger) and observing whether the hydrogel remained attached without falling.

#### Self‑healing ability

Two disc‑shaped hydrogel samples were stained with red and blue food dyes, respectively. Each sample was cut into halves, and the differently colored halves were placed in contact. After 1 min, the rejoined hydrogel was gently pulled from both sides with forceps to demonstrate its structural integrity.

#### Surface morphology by SEM

Lyophilized hydrogel samples underwent gold sputter-coating prior to imaging with a scanning electron microscopy (SEM (JSM7100F, Japan) to examine their internal porous architecture.

#### Chemical analysis by FTIR spectroscopy

Fourier‑transform infrared (FTIR) spectra of CS, lyophilized PVA/Col and PVA/Col/CS hydrogel were recorded over the 4000–500 cm^−1^ range to verify component integration and boronate ester bond formation.

#### Swelling and degradation profiles

For swelling tests, pre-weighed dry hydrogels were submerged in PBS and maintained at 37°C. The swollen weight was recorded periodically until equilibrium. The swelling ratio, expressed as (Wₛ-W_0_) divided by W_0_, was quantified. Degradation was evaluated under two conditions: (i) hydrolysis in sterile PBS and (ii) enzymatic degradation in PBS containing 1 U mL^−1^ collagenase I and Ⅱ. The remaining mass was measured periodically over 10 days, and the mass loss percentage was calculated.

#### Rheological characterization

Rheological properties were measured using a rotational rheometer (AR2000Ex, TA Instruments) with a parallel-plate geometry. Self-healing: A step-strain test alternated oscillatory strain between 1% (low, within linear viscoelastic region) and 100% (high) at 1 Hz to monitor the recovery of storage (G′) and loss (G″) moduli over cycles. Shear-thinning: A steady-state flow test measured viscosity over a shear rate range of 0.1–100 s^−1^. Gelation kinetics: A time-sweep test at 1% strain and 1 Hz frequency monitored the evolution of G′ and G″ at 37°C for 15 min to confirm gel formation.

### Rat IVDD model establishment

#### Surgical induction of disc degeneration and hydrogel injection

All animal procedures were approved by the Animal Ethics Committee of Soochow University (SUDA20240711A10). Under isoflurane anesthesia and aseptic conditions, disc degeneration was induced in the Co7/8 caudal disc of rats by puncturing with a 20-gauge needle, rotating 360°, holding for 1 min and partially removing NP tissue. Immediately post-puncture, rats were randomly allocated to receive an injection of 5 µL of either PBS (IVDD control) or one of the hydrogel formulations (PVA/Col, PVA/Col/CS-A, PVA/Col/CS-C, PVA/Col/CS-A&C) into the same disc using a 25-gauge needle. The wound was then closed.

#### Imaging evaluation

Animals underwent euthanasia at 4 and 8 weeks post-surgery, with subsequent retrieval of caudal spinal columns. The Disc Height Index (DHI) was determined from X-ray images. T2-weighted MRI was performed to assess nucleus pulposus signal intensity and to grade disc degeneration according to the Pfirrmann classification (grades I-V). All MRI and X-ray assessments were performed by two independent observers who were blinded to the experimental group assignments. The Pfirrmann grade was determined in a blinded manner.

#### Histological processing and analysis

Harvested caudal spines were sagittally sectioned for macroscopic observation. After imaging, segments were first fixed in 4% paraformaldehyde and then subjected to decalcification in 10% EDTA for 2 months and processed for paraffin embedding. Sagittal sections underwent H&E and Safranin O-Fast Green staining (5 µm thickness). Disc degeneration was scored using an established histological grading system. All histological and immunohistochemical evaluations were conducted by two independent observers blinded to the treatment groups. IHC for CS (Abcam, ab11570), COL2A1 (Abcam, ab34712) and SOD1 (Abcam, ab51254) was performed using specific primary and secondary antibodies with DAB development. The percentage of positive area (CS, COL2A1) or positive cells (SOD1) was quantified using ImageJ software.

### Statistics analysis

All quantitative data are presented as the mean ± standard deviation (SD). Statistical analyses were performed using GraphPad Prism software (version 9.2). For comparisons between two groups, an unpaired two-tailed Student’s *t*-test was applied. For comparisons among multiple groups, one-way or two-way analysis of variance (ANOVA) was employed, followed by Tukey’s post-hoc test for multiple comparisons. For correlation analyses involving ordinal data or non-normally distributed variables, Spearman’s rank-order correlation coefficient was calculated to assess the strength and direction of monotonic relationships. The sample size (*n*) for each experiment is specified in the corresponding figure legend. A *P* value of less than 0.05 was considered statistically significant. Specific statistical methods for histological/MRI grading are detailed in their respective experimental subsections.

## Results

### The association between depleted CS levels and IVDD: evidence from human tissues to rat models

**Figure 2:**
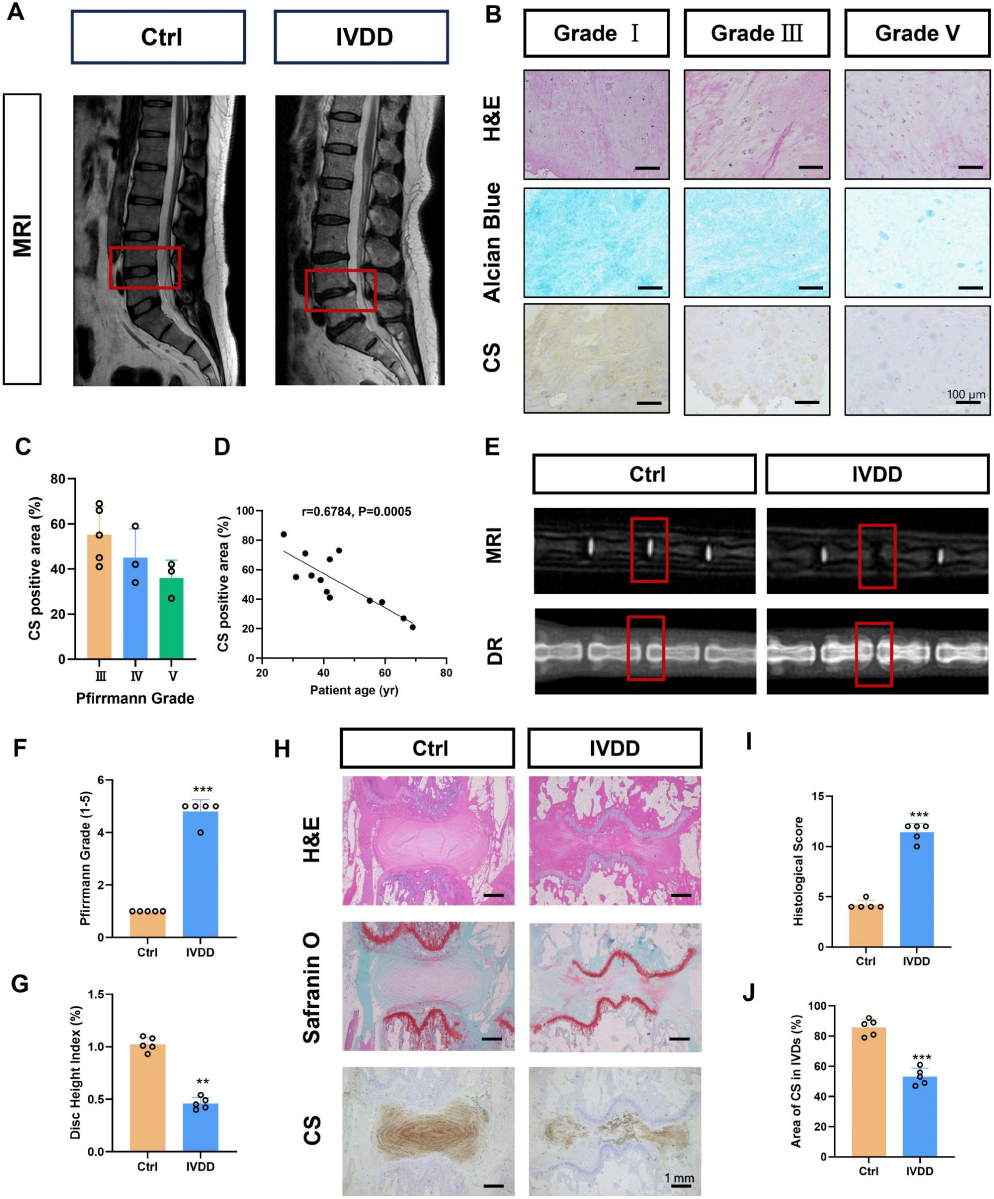
Expression of GAGs and CS in human and rat IVD. (**A**) T2-weighted MRI images of human IVD (red wireframe indicate the corresponding IVD). (**B**) H&E, alcian blue and immunohistochemical staining of human IVD (scale bar = 100 μm). (**C**) Quantification of the rates of CS-positive area. (**D**) Spearman’s rank-order correlation coefficient of CS expression levels with Pfirrmann grade of donor sources. (**E**) Representative T2-weighted MRI and X-ray images of caudal intervertebral discs from the control and IVDD groups. (**F**) Quantitative analysis of Pfirrmann grading scores based on MRI. (**G**) Quantitative analysis of the DHI based on X-ray images. (**H**) Representative histological sections stained with H&E, Safranin O-Fast Green (S-O) and IHC for CS. (**I**) Quantitative histological scores of the intervertebral discs. (**J**) Quantitative analysis of CS content based on IHC staining. Data in (**F, G, I, J**) are presented as mean ± SD (*n* = 5). **P < 0.01, ***P < 0.001 vs. Control group.

In order to initially clarify the changes of CS in IVDD, we first evaluated the expression of CS in degenerated IVD tissues acquired from patients receiving spinal surgical procedures (Figure 2A).

Alcian blue stain showed significant decrease of total GAG (Figure 2B). Similarly, immunohistochemical analysis revealed that CS-positive area reduced in severe degeneration (Pfirrmann grade Ⅴ) (Figure 2B). In addition, CS positivity is negatively correlated with the Pfirrmann grade and age (Figure 2C and D). As illustrated in Figure 2E, representative MRI and X-ray images revealed notable structural deterioration in the degenerated rat caudal discs relative to the controls. Quantitatively, the degeneration group exhibited a significant increase in Pfirrmann grade (Figure 2F), accompanied with a marked drop in the DHI, indicating severe disc space narrowing (Figure 2G). Histological examination further corroborated these findings. H&E and Safranin O-Fast Green staining, combined with immunohistochemical staining for CS, demonstrated a collapsed disc structure, blurred boundary between the NP and AF, and a substantial loss of CS in the degenerated discs (Figure 2H). Consistent with these observations, quantitative histological analysis revealed a significantly higher score (Figure 2I), while a significant reduction in CS content was observed in the degeneration group when compared with controls (Figure 2J).

### CS promotes anabolism and antioxidant enzymes in rat AFCs and NPCs

**Figure 3:**
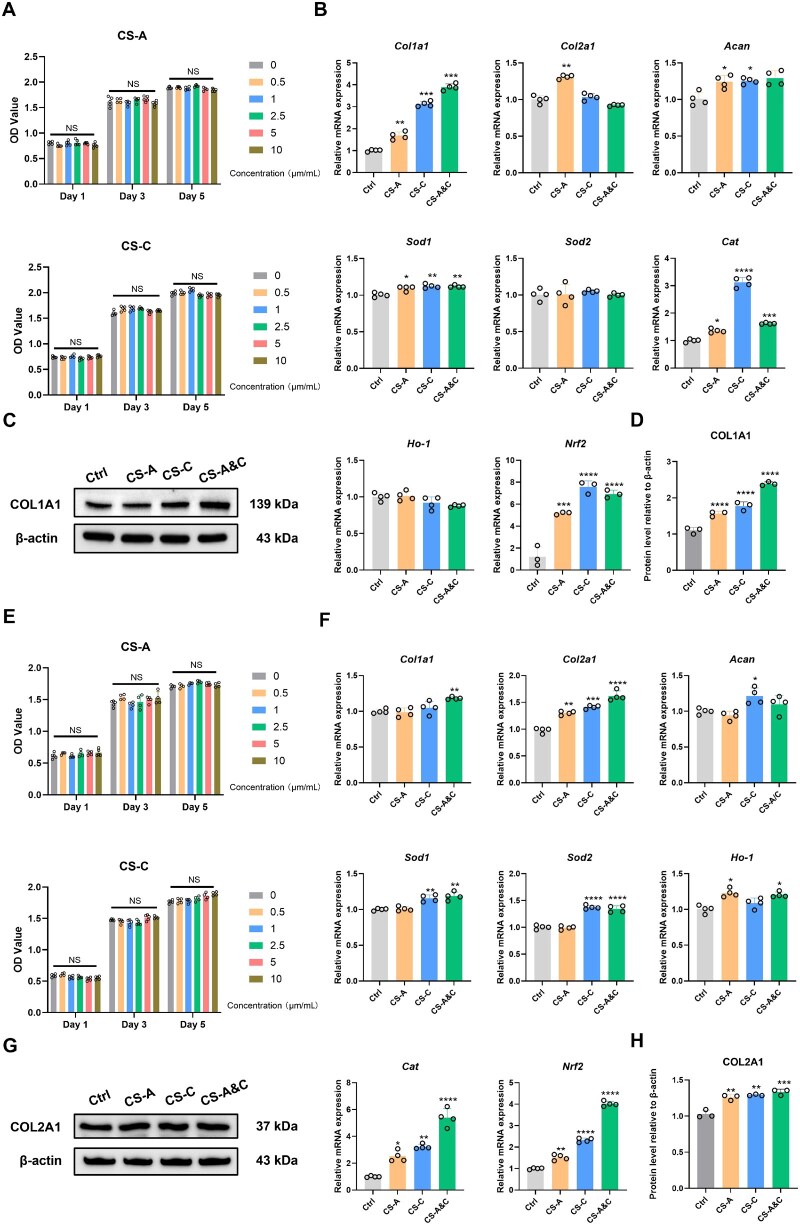
The effects of CS on the anabolism and antioxidant enzymes of AFCs and NPCs by adding it to the culture medium. NPCs and AFCs were treated with CS-A at a concentration of 100 μg mL^−1^, CS-C at a concentration of 100 μg mL^−1^, or CS-A and CS-C at a concentration of 50 μg mL^−1^. (**A**) Cell proliferation of rat AFCs after treatment with CS-A or CS-C was examined at 1, 3 and 5 days using CCK-8 assay, *n* = 5. (**B**) Relative mRNA expression levels of *Col1a1*, *Col2a1*, *Acan*, *Sod1*, *Sod2*, *Ho-1*, *Cat* and *Nrf2* in AFCs following treatment with CS, *n* = 4. (**C**) Western blot for COL1A1 representing a key ECM component in AFCs, *n* = 3. (**D**) Quantification of COL1A1 protein expression levels relative to β-actin. (**E**) Cell proliferation of rat NPCs after treatment with CS-A or CS-C was examined at 1, 3 and 5 days using CCK-8 assay, *n* = 5. (**F**) Relative mRNA expression levels of *Col1a1*, *Col2a1*, *Acan*, *Sod1*, *Sod2*, *Ho-1*, *Cat* and *Nrf2* in NPCs following treatment with CS, *n* = 4. (**G**) Western blot for COL2A1 representing a key ECM component in NPCs, *n* = 3. (**H**) Quantification of COL2A1 protein expression levels relative to β-actin. Data are presented as mean ± SD; *P < 0.05, **P < 0.01, ***P < 0.001, ****P < 0.0001 vs. Control group, NS, not significant.

To assess how CS subtypes affect cell viability, we added a gradient of concentrations of the CS subtypes to the culture medium of NPCs and AFCs respectively and cultured for 3 days. CCK-8 assays showed good biocompatibility and no obvious cytotoxicity of CS (Figure 3A and E).

To explore the potential impact of CS on intervertebral disc cells, we evaluated the ECM anabolic gene expression of CS treated AFCs and NPCs. RT-PCR analysis revealed that CS-A&C upregulated Col1a1 of AFCs and Col2a1 of NPCs by 3.9-fold and 1.6-fold, with a slight increase in Acan (Figure 3B and F). Western blot further confirmed that a 2.4-fold increase of COL1A1 in AFCs (Figure 3C and D) and a 1.3-fold increase of COL2A1 in NPCs (Figure 3G and H). Furthermore, we observed that CS treatment increased the transcript abundance of key antioxidant genes, namely Sod1, and Cat in AFCs, Sod1, Sod2, Cat and Ho-1 in NPCs, with a 6.9-fold and 3.9-fold upregulation in Nrf2 respectively (Figure 3B and 2F).

### CS alleviates oxidative stress and restores matrix homeostasis by enhancing intracellular antioxidative system in AFCs

**Figure 4:**
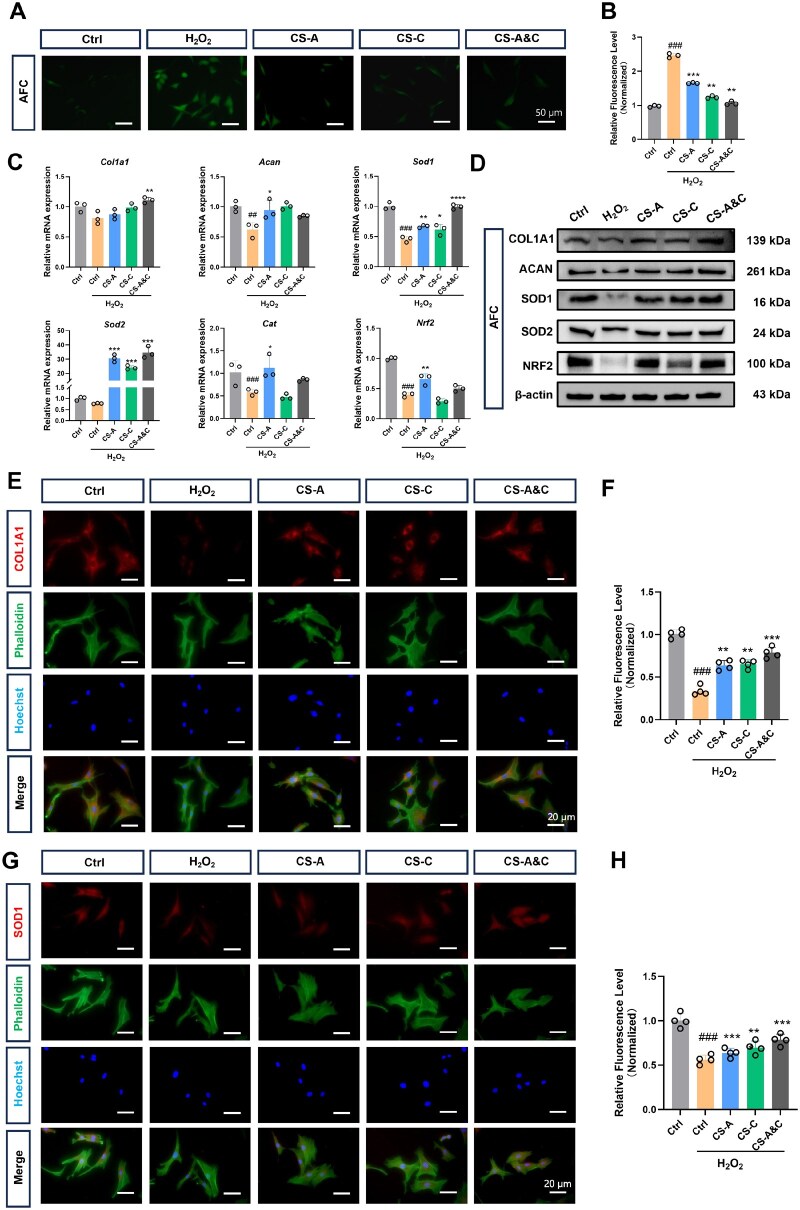
CS restores matrix homeostasis by enhancing intracellular antioxidative system in AFCs. (**A**) Representative fluorescent images of intracellular ROS levels detected by DCFH-DA staining (scale bar: 50 μm). (**B**) Quantitative analysis of relative fluorescence intensity. AFCs were exposed to 200 μM H_2_O_2_ for 2 h, followed by treatment with CS subtypes (CS-A, CS-C or CS-A&C) for 3 days. (**C**) mRNA expression levels of ECM-related genes (*Col1a1, Acan*) and key antioxidant genes (*Sod1, Sod2, Cat*) and the regulator *Nrf2* in AFCs following H_2_O_2_ stimulation with CS treatment, as determined by qRT-PCR. (**D**) Representative Western blot images of the proteins encoded by the above-mentioned genes. (**E**) Representative immunofluorescence images showing COL1A1 (red) in AFCs. The cytoskeleton (F-actin) and nuclei are counterstained with Phalloidin (green) and Hoechst (blue), respectively (scale bar: 20 μm). (**F**) Quantitative analysis of COL1A1 fluorescence intensity corresponding to (**E**). (**G**) Representative immunofluorescence images showing SOD1 (red) under the same experimental conditions (scale bar: 20 μm). (**H**) Quantitative analysis of SOD1 fluorescence intensity corresponding to (**G**). Data are presented as mean ± SD; ^#^*P* < 0.05, ^##^*P* < 0.01, ^###^*P* < 0.001, ^####^*P* < 0.0001 vs. Control group, *P < 0.05, **P < 0.01, ***P < 0.001, ****P < 0.0001 vs. Degeneration group.

To examine the cytoprotective role of CS under conditions of oxidative stress, we first successfully generated an *in vitro* oxidative stress model through exposure of AFCs to hydrogen peroxide (H_2_O_2_) to mimic the process of IVDD. DCFH-DA staining confirmed a substantial accumulation of intracellular ROS in the H_2_O_2_-treated group (Figure 4A), which was accompanied by a significant suppression of cell proliferation (Supplementary Figure S1A).

In contrast, treatment with CS subtypes for 3 days markedly attenuated this H_2_O_2_-induced ROS generation. Subsequent analysis by RT-PCR revealed that CS effectively rescued the impairment of *Col1a1* and *Acan* caused by oxidative stress. Notably, the combined treatment of CS-A&C exhibited the most potent effect in restoring the expression of matrix-related genes (Figure 4C). Furthermore, CS significantly bolstered the cellular antioxidant genes including *Sod1*, *Sod2* and *Nrf2* under oxidative stress conditions (Figure 4C). Relative to the oxidative stress group, the CS-A&C group showed a pronounced increase in antioxidant enzyme protein expression by Western blot (Figure 4D; Supplementary Figure S1C). Compared to gene expression levels, these restored protein levels approximated control group levels, a phenomenon that may be attributed to post-transcriptional and translational regulation. Through immunofluorescence staining, we can intuitively observe that the oxidative stress group exhibited a marked decrease in COL1A1, while the CS subtype groups showed varying degrees of increase. The CS-C subtype was primarily localized nearer to the nucleus, whereas the CS-A&C group demonstrated a uniform distribution throughout the cytoplasm (Figure 4E and F). Similarly, the SOD1 fluorescence intensity decreased by roughly half in the H_2_O_2_ group, while it increased to approximately 70% in the CS-A&C groups (Figure 4G and H).

### CS alleviates oxidative stress and restores matrix homeostasis by enhancing intracellular antioxidative system in NPCs

**Figure 5:**
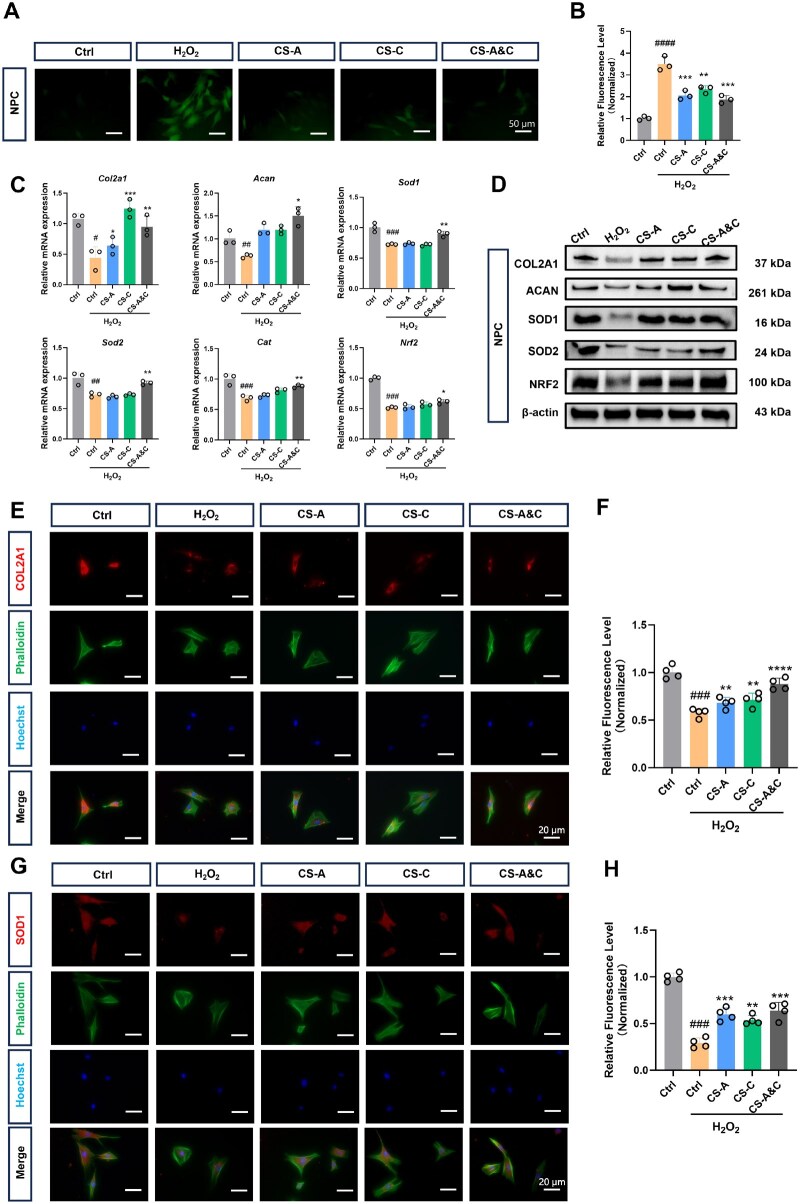
CS restores matrix homeostasis by enhancing intracellular antioxidative system in NPCs. (**A**) Representative fluorescent images of intracellular ROS levels detected by DCFH-DA staining (scale bar: 50 μm). (**B**) Quantitative analysis of relative fluorescence intensity. NPCs were exposed to 200 μM H_2_O_2_ for 2 h, followed by treatment with CS subtypes (CS-A, CS-C or CS-A&C) for 3 days. (**C**) mRNA expression levels of ECM-related genes (*Col2a1, Acan*) and key antioxidant genes (*Sod1, Sod2, Cat*) and the regulator *Nrf2* in NPCs following H_2_O_2_ stimulation with CS treatment, as determined by qRT-PCR. (**D**) Representative Western blot images of the proteins encoded by the above-mentioned genes. (**E**) Representative immunofluorescence images showing COL2A1 (red) in NPCs. The cytoskeleton (F-actin) and nuclei are counterstained with Phalloidin (green) and Hoechst (blue), respectively (scale bar: 20 μm). (**F**) Quantitative analysis of COL2A1 fluorescence intensity corresponding to (**E**). (**G**) Representative immunofluorescence images showing SOD1 (red) under the same experimental conditions (scale bar: 20 μm). (**H**) Quantitative analysis of SOD1 fluorescence intensity corresponding to (**G**). Data are presented as mean ± SD; ^#^*P* < 0.05, ^##^*P* < 0.01, ^###^*P* < 0.001, ^####^*P* < 0.0001 vs. Control group, *P < 0.05, **P < 0.01, ***P < 0.001, ****P < 0.0001 vs. Degeneration group.

Likewise, we utilized H_2_O_2_ to induce an oxidative stress model in NPCs (Supplementary Figure S1B). We observed a widespread and uniform distribution of the DCFH-DA probe within the cells of the stress group. Intracellular ROS were significantly reduced in both the CS-A group and the CS-A&C group (Figure 5A and B).

The expression of *Col2a1* and *Acan* genes were effectively rescued under oxidative stress conditions in the CS-A&C group (Figure 5C). Western blotting corroborated that the levels of the encoded proteins were correspondingly elevated (Figure 5D; Supplementary Figure S1D). Although the upregulation of *Sod1*, *Sod2*, *Cat* and *Nrf2* genes relative to the degeneration group was exclusive to the combination group, all CS subtype groups exhibited an increase in the levels of the target proteins (Figure 5C and D; Supplementary Figure S1D). Immunofluorescence analysis revealed that oxidative stress induced a noticeable shrinkage and rounding of NPCs, accompanied by a marked reduction in the fluorescence intensity of both COL2A1 and SOD1 (Figure 5E–H). Treatment with CS subtypes demonstrated an ameliorative effect on these stress-induced morphological alterations and concurrently enhanced the fluorescence intensity of both proteins (Figure 5E–H).

### CS protects AFCs against degeneration by preserving mitochondrial integrity

**Figure 6:**
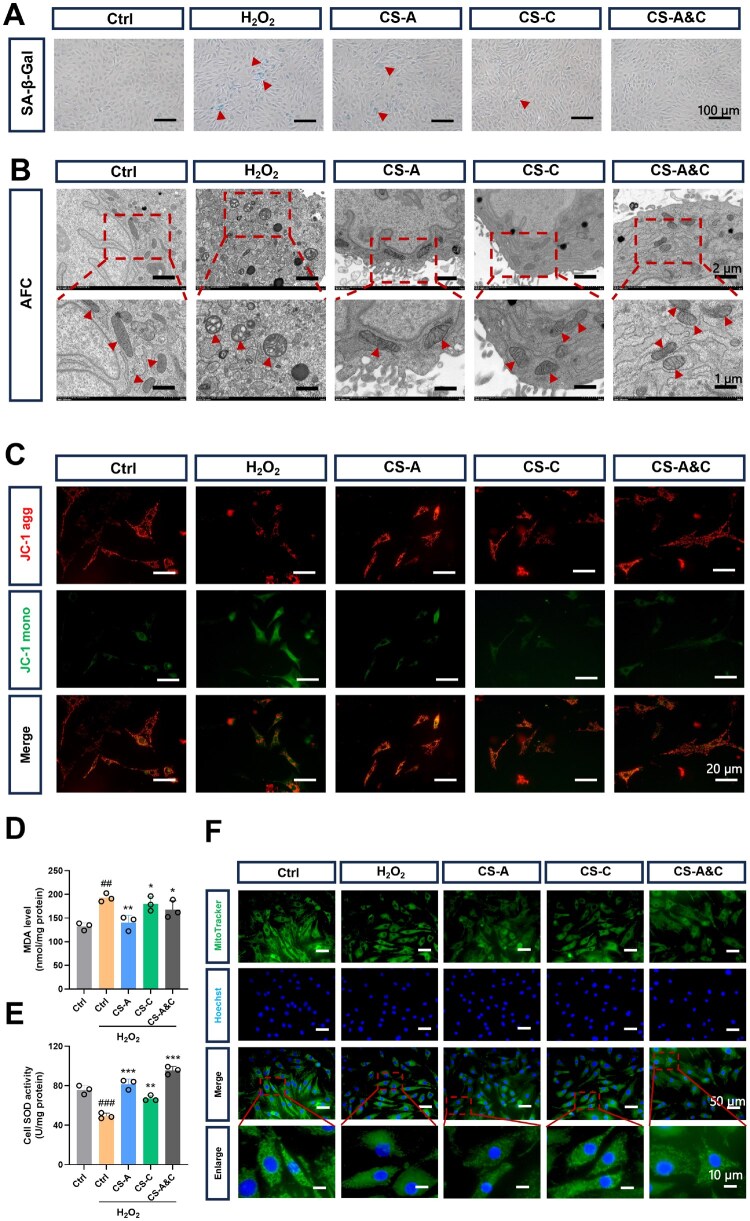
CS reduces oxidative stress and preserves mitochondrial integrity in AFCs. (**A**) SA-β-galactosidase staining of senescent cells. Blue staining indicates increased senescence-associated β-galactosidase activity (scale bar: 100 μm). (**B**) TEM images of mitochondrial ultrastructure (scale bar: 2 μm and 1 μm). (**C**) JC-1 fluorescence staining assessing mitochondrial membrane potential. Red fluorescence (J-aggregates) indicates high membrane potential, while green fluorescence (J-monomers) indicates depolarization (scale bar: 20 μm). Quantitative analysis of (**D**) MDA content and (**E**) total SOD activity. (**F**) Mitochondrial network morphology visualized by MitoTracker (green) staining. Nuclei are counterstained with Hoechst (blue). Red wireframes show higher-magnification views. AFCs were treated with CS subtypes after H_2_O_2_ exposure. Data in (**D**) and (**E**) are presented as mean ± SD. ^##^*P* < 0.01, ^###^*P* < 0.001 vs. Control group, *P < 0.05, **P < 0.01, ***P < 0.001 vs. Degeneration group.

To further explore the underlying mechanism through which CS mediates its rescue effect on disc degeneration, we characterized the alterations in mitochondrial morphology and function. SA-β-Gal staining demonstrated that CS-A, CS-C and CS-A&C rescued ROS-induced AFCs senescence and degeneration (Figure 6A; Supplementary Figure S2A).

Next, transmission electron microscopy (TEM) revealed that untreated AFCs exhibited tubular mitochondria of varying lengths. In the oxidative stress group, the mitochondria were markedly swollen and displayed internal vacuolization, accompanied by disrupted cristae structure and reduced electron density. The CS-A group presented elongated, mildly swollen tubular mitochondria, whereas the CS-C group featured short, rod-shaped mitochondria with reappearance of functional cristae structures. In the CS-A&C group, mitochondrial size and morphology closely resembled those of the control group, which exhibited significantly improved cristae density and clarity (Figure 6B). The mitochondrial membrane potential can be measured by JC-1 staining. A shift in fluorescence from red to green signifies mitochondrial depolarization. CS-C and CS-A&C groups significantly suppressed the decline in oxidative stress-induced mitochondrial membrane potential (Figure 6C; Supplementary Figure S2C). Our results demonstrated that CS-A significantly reduced the MDA level in AFCs under oxidative stress conditions (Figure 6D). Correspondingly, CS subtypes effectively enhanced the expression of total SOD in these cells (Figure 6E). Furthermore, using MitoTracker staining, we observed elongated tubular mitochondrial networks in the controls. In contrast, the H_2_O_2_ group exhibited fragmented, granular mitochondria accompanied by a marked decline in fluorescence intensity. Conversely, the mitochondrial morphology and fluorescence intensity in both the CS-C and CS-A&C groups closely resembled those of the control group (Figure 6F; Supplementary Figure S2E).

### CS protects NPCs against degeneration by preserving mitochondrial integrity

**Figure 7:**
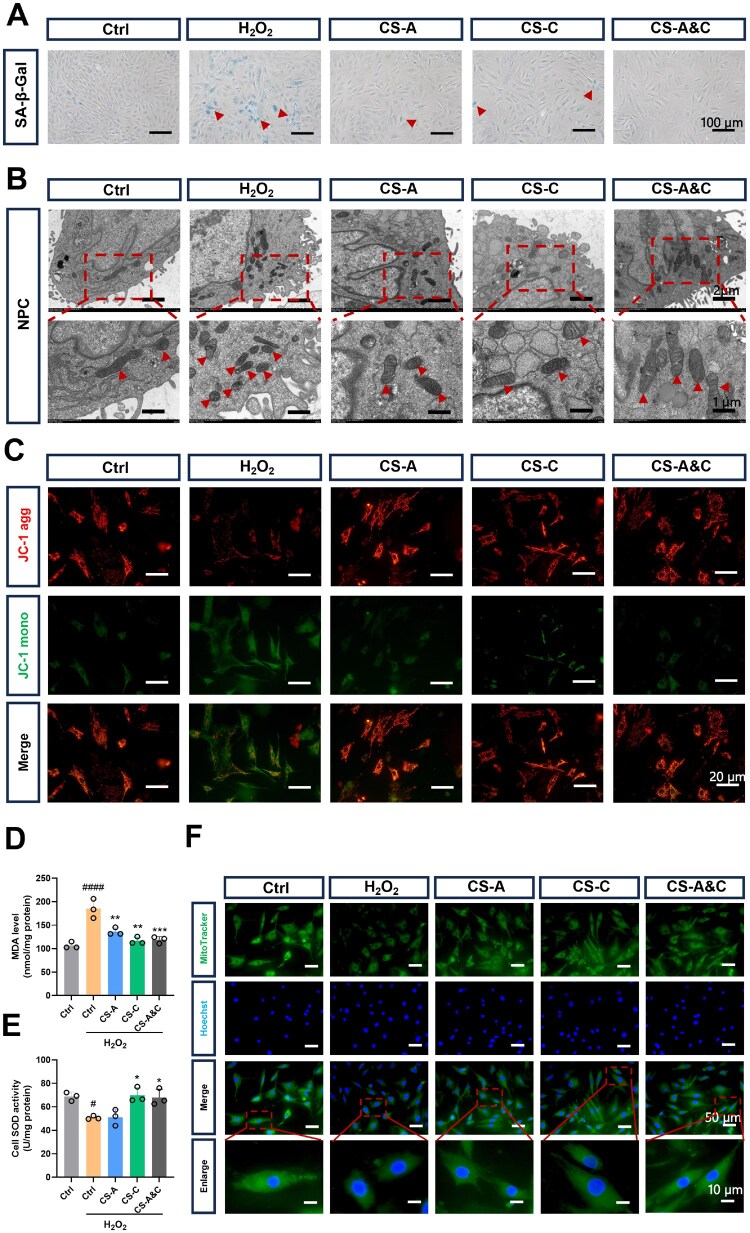
CS reduces oxidative stress and preserves mitochondrial integrity in NPCs. (**A**) SA-β-galactosidase staining of senescent cells. Blue staining indicates increased senescence-associated β-galactosidase activity (scale bar: 100 μm). (**B**) TEM images of mitochondrial ultrastructure (scale bar: 2 μm and 1 μm). (C) JC-1 fluorescence staining assessing mitochondrial membrane potential. Red fluorescence (J-aggregates) indicates high membrane potential, while green fluorescence (J-monomers) indicates depolarization (scale bar: 20 μm). Quantitative analysis of (**D**) MDA content and (**E**) total SOD activity. (**F**) Mitochondrial network morphology visualized by MitoTracker (green) staining. Nuclei are counterstained with Hoechst (blue). Red wireframes show higher-magnification views. NPCs were treated with CS subtypes after H_2_O_2_ exposure. Data in (**D**) and (**E**) are presented as mean ± SD. ^#^*P* < 0.05, ^####^*P* < 0.0001 vs. Control group, *P < 0.05, **P < 0.01, ***P < 0.001 vs. Degeneration group.

Similarly, we assess the protective role of CS on mitochondria in NPCs. Firstly, SA-β-Gal staining revealed oxidative stress-induced cellular senescence and a marked suppression of proliferation in these cells (Figure 7A; Supplementary Figure S2B).

Subsequently, TEM observations demonstrated that the degeneration group exhibited shrunken mitochondria with reduced and shortened cristae, alongside increased electron density. Both CS-A group and CS-C group improved cristae integrity and attenuated mitochondrial shrinkage, while the elevated electron density was also effectively alleviated in the CS-A&C group (Figure 7B). Regarding mitochondrial function, the CS subtypes significantly increased the membrane potential levels, with the CS-A&C group reaching approximately 75% of the control level (Figure 7C; Supplementary Figure S2D). Furthermore, we found that CS-C and CS-A&C comparably suppressed MDA production and rescued total intracellular SOD enzyme activity (Figure 7D and E). Mitotracker staining revealed that all H_2_O_2_-stimulated groups exhibited mitochondria with indistinct, granular morphology scattered throughout the cytoplasm. However, the CS-A&C group demonstrated the strongest fluorescence intensity, approximately 20% higher than that of either the CS-A or CS-C groups alone (Figure 7F; Supplementary Figure S2F).

### Exploring the mechanism underlying the therapeutic action of CS on disc degeneration

**Figure 8:**
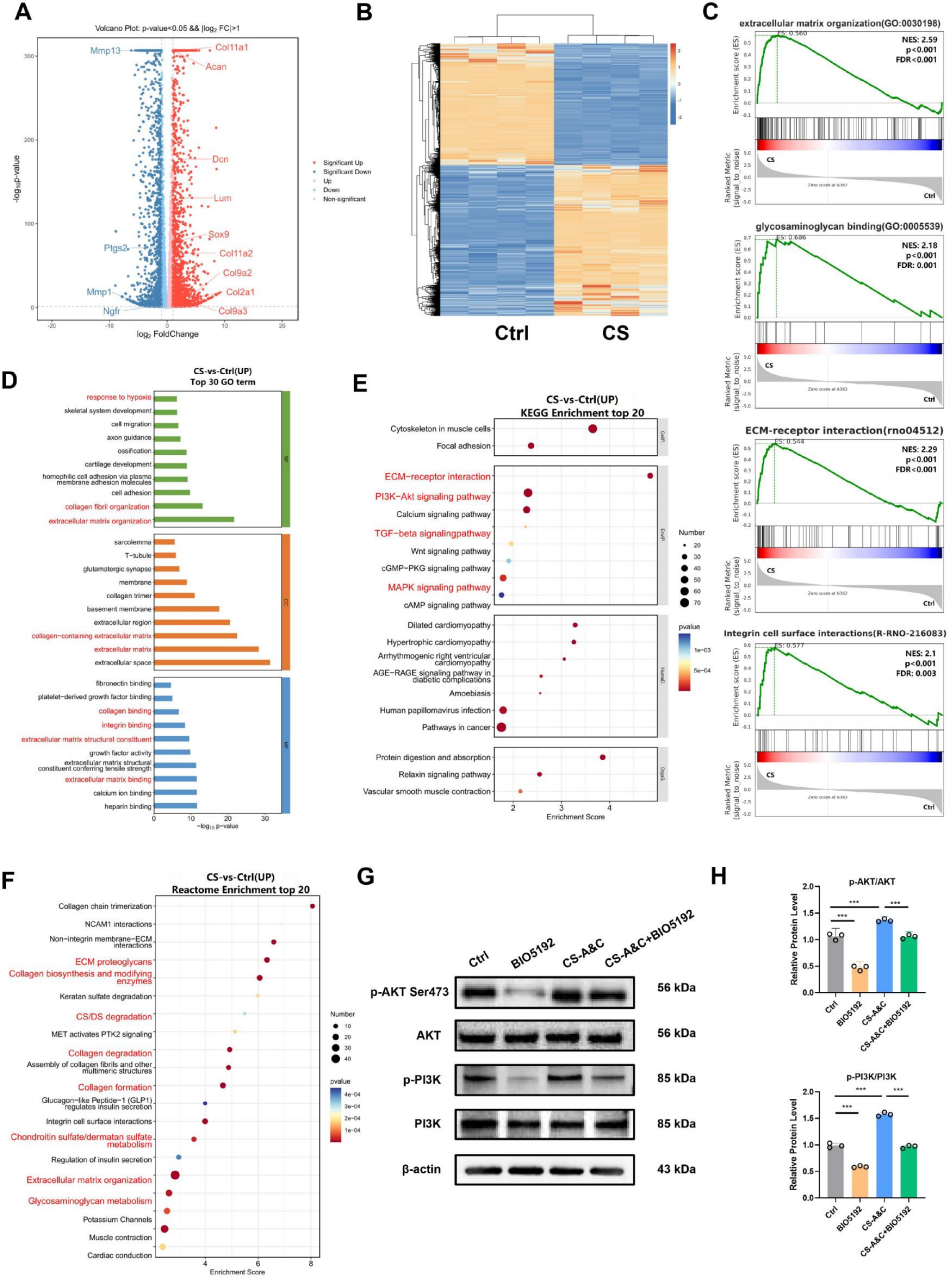
Transcriptomic profiling and validation reveal that CS activates the integrin-PI3K-Akt signaling pathway in NPCs. (**A**) Volcano plot displaying DEGs between CS-treated and control groups. (**B**) Heatmap of the expression patterns of the most significant DEGs. (**C**) GSEA plot showing significant enrichment of a gene set related to ECM organization, ECM-receptor interaction, GAG binding and integrin cell surface interactions. (**D**) GO enrichment analysis of DEGs, categorized into biological process (BP), cellular component (CC) and molecular function (MF). (**E**) KEGG pathway enrichment analysis. (**F**) Reactome pathway enrichment analysis. (**G**) Representative Western blots of key proteins in the PI3K-Akt pathway, including the phosphorylated (p-PI3K, p-AKT) and total (PI3K, AKT) forms. (**H**) Quantitative analysis of the protein phosphorylation levels from (**G**), presented as the ratios of p-PI3K/PI3K and p-AKT/AKT. NPCs were treated with CS for 3 days for RNA sequencing (**A-F**) and Western blot (**G, H**) analyses. Data in (**H**) are presented as mean ± SD (*n* = 3). ***P < 0.001 vs. Control group.

To elucidate the mechanism by which CS maintains the matrix-synthetic phenotype and mitochondrial integrity (in both morphology and function) of intervertebral disc cells, we performed transcriptome sequencing following CS intervention in NPCs. The volcano plot revealed 1873 significantly upregulated genes and 1508 significantly downregulated genes. Among these, upregulated matrix synthesis-related genes included *Acan*, *Col2a1*, *Sox9*, *Lum* and *Dcn*, while downregulated matrix degradation-related genes included, for example, *Mmp1* and *Mmp13* (Figure 8A).

GO analysis further revealed that the DEGs were enriched in biological processes such as the response to hypoxia, collagen fibril organization and ECM organization; in cellular components including the collagen-containing ECM and ECM; and in molecular functions such as collagen binding and integrin binding (Figure 8D). KEGG pathway analysis demonstrated significant upregulation of signaling pathways including the ECM-receptor interaction, the PI3K-Akt signaling pathway, TGF-beta signaling pathway and MAPK signaling pathway (Figure 8E). Furthermore, Reactome pathway enrichment suggested upregulation in pathways related to ECM proteoglycans, ECM organization and GAG metabolism. Notably, pathways for CS degradation and CS metabolism, as well as collagen formation and degradation, were concurrently upregulated (Figure 8F). Gene set enrichment analysis (GSEA) further validated an increase in the activity of signaling pathways linked to ECM organization, ECM-receptor interaction, GAG binding and integrin cell surface interactions (Figure 8C).

Transcriptomic profiling of CS-treated AFCs further corroborated its role in mitochondrial regulation. GO enrichment analysis of the DEGs revealed marked upregulation in pathways associated with peroxidase activity and the mitochondrial respiratory chain complex V (Supplementary Figure S3A). Subsequent GSEA confirmed a marked enrichment for gene sets associated with mitochondrial respiratory chain complex V (Supplementary Figure S3B), supporting the notion that CS enhances mitochondrial oxidative phosphorylation and redox homeostasis in AF cells.

Based on an integrated analysis of upregulated pathways in KEGG and pathways closely associated with ECM synthesis and mitochondrial function in NPCs, we postulated that CS mitigates IVDD via the PI3K-Akt signaling pathway. To investigate its activation status, we measured PI3K, AKT and their phosphorylated forms protein levels. Western blotting results demonstrated that CS markedly increased the phosphorylation of both PI3K and AKT (Figure 8G and H). The activation of the PI3K-AKT pathway by CS, mediated through integrin α4β1 binding, has been reported, so we introduced BIO-5192, a specific inhibitor of integrin α4β1. The results demonstrated that BIO-5192 caused a significant reduction in the phosphorylation of both PI3K and AKT (Figure 8G and H). Collectively, these findings identify that CS regulates mitochondrial function and morphological integrity, and promotes ECM synthesis in NPCs through the integrin α4β1-PI3K-Akt signaling axis.

### Fabrication and characterization of a bioinspired ECM hydrogel loaded with CS

**Figure 9:**
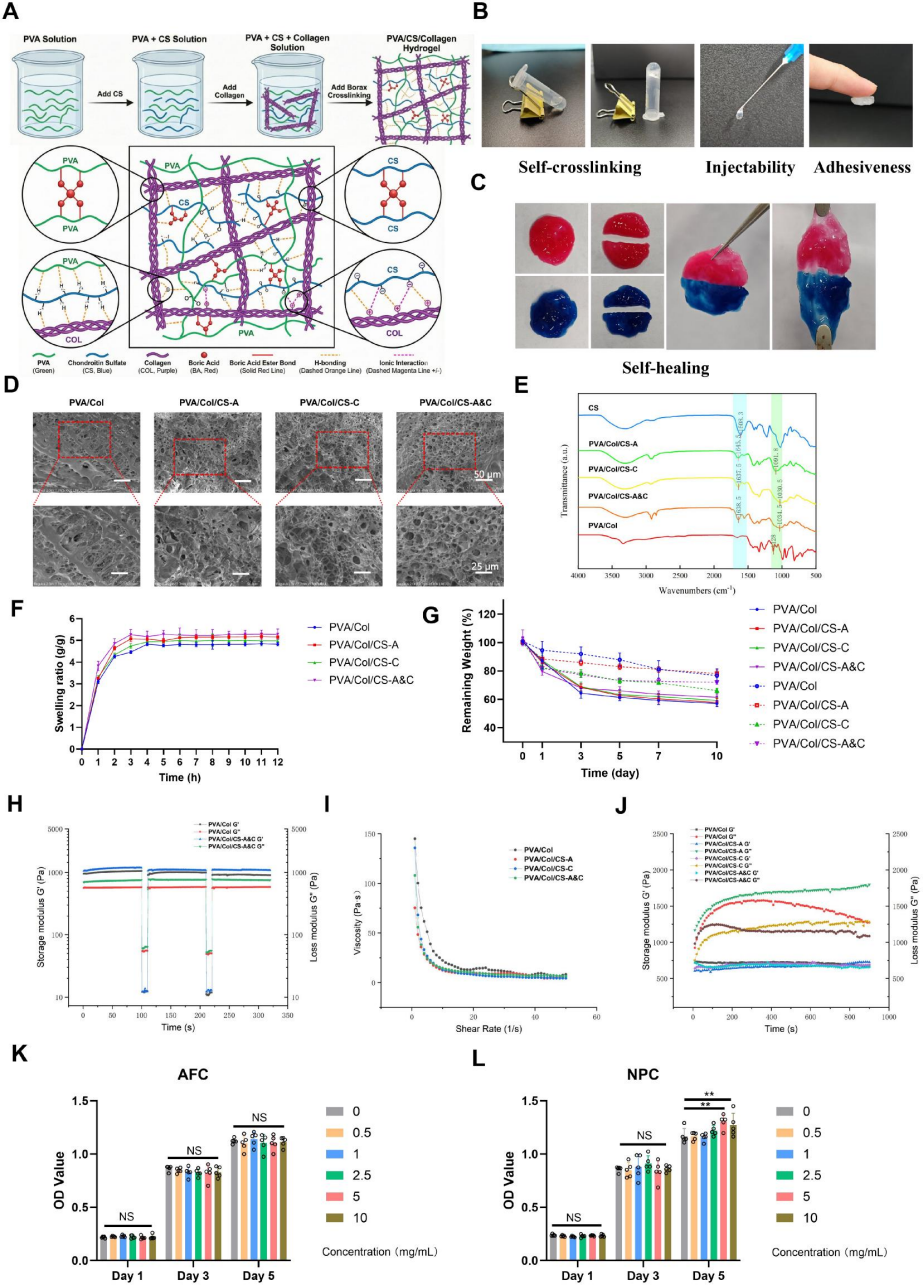
Comprehensive characterization of the bioinspired ECM hydrogel loaded with CS. (**A**) Schematic illustration of the fabrication and the crosslinked network structure of the PVA/Col/CS hydrogel. The network is formed through dynamic boronic ester bonds between boric acid and the cis-diol groups on both PVA and CS chains, while collagen is incorporated within the network to provide bioactivity. (**B**) Schematic illustration of hydrogel formation via the inversion method. Photographs demonstrating the injectability and adhesive properties of the hydrogel. (**C**) Self-healing behavior observed after cutting, rejoining and stretching the hydrogel. (**D**) SEM images of hydrogels without CS and with different CS subtypes (scale bars: 50 μm and 25 μm). (**E**) FTIR spectra of CS and the fabricated hydrogels. (**F**) Swelling kinetics of the hydrogel in PBS. (**G**) In vitro degradation profiles of the hydrogel via hydrolysis (in PBS) and enzymatic degradation (in collagenase solution). rheological characterization: (**H**) Step-strain cycle tests (strain: 1% → 100% → 1%) demonstrating self-recovery. (**I**) Steady-state shear viscosity measurements showing shear-thinning behavior. (**J**) Time-sweep test at 37°C confirming gelation. CCK-8 assays quantifying the proliferation of (**K**) AFCs and (**L**) NPCs after 1, 3 and 5 days of culture with hydrogel extracts. Data in (**K**) and (**L**) are presented as mean ± SD (*n* = 5). **P < 0.01, NS, not significant.

Within the field of IVD regeneration, the application of tissue engineering strategies, such as hydrogels, holds promise for repairing and even regenerating damaged disc tissues, thereby restoring their mechanical function and biological activity. Based on this, we synthesized a hybrid hydrogel platform comprising three key components: mechanically adaptive PVA, biomimetic collagen representing the ECM, and CS which targets the regulation of matrix synthesis and mitochondrial antioxidant functions in disc cells. Within this system, PVA primarily serves to simulate the mechanical properties of the AF or to support the NP in withstanding axial compression. Collagen is incorporated to provide bioactive cues that promote cell adhesion, proliferation and differentiation, thereby mimicking the native ECM. After mixing with boric acid, the system can crosslink to form a hydrogel (Figure 9A).

Its excellent injectability allows the hydrogel to be extruded through a 22-gauge needle, and the crosslinked hydrogel can adhere to the skin surface (Figure 9B). Two separately cut semicircular hydrogel pieces can rejoin into a complete circle within 1 min and withstand stretching (Figure 9C). SEM images reveal a clear three-dimensional porous network within the hydrogel (Figure 9D). FTIR spectroscopy confirmed the presence of characteristic peaks for both CS and boronic ester bonds in the hydrogel system (Figure 9E). The swelling test results indicated that the hydrogel reached an equilibrium swelling ratio of 5 g g^−1^ in PBS within 6 h (Figure 9F).

Regarding its degradation profile, the hydrogel demonstrated excellent stability during hydrolysis in sterile PBS, with only 20% mass loss over 10 days. In contrast, it exhibited significantly accelerated degradation in collagenase-containing solution, achieving approximately 40% degradation within the same period (Figure 9G). Alternating strain step tests revealed that upon changes in strain, the values of G′ and G″ exhibited corresponding inversions, and after multiple cycles, the hydrogel was still able to largely recover, thereby demonstrating its excellent self-healing capability (Figure 9H). Steady-state flow tests indicated that the apparent viscosity of the hydrogel dropped sharply from approximately 150 Pa·s at a low shear rate (0.1 s^−1^) to about 4 Pa·s under high shear rate (50 s^−1^), further confirming its pronounced shear-thinning behavior (Figure 9I). Frequency sweep results showed that over the entire tested frequency range, G′ consistently remained more than an order of magnitude higher than G″, and both modulus curves were relatively flat, indicating the successful formation of a stable, covalently crosslinked hydrogel with typical solid-like behavior (Figure 9J). Subsequent live/dead staining and CCK-8 assays using both NPCs and AFCs confirmed the hydrogel’s outstanding biocompatibility (Figure 9K and L; Supplementary Figure S4A and B). Hydrogels loaded with different CS subtypes were pre‑stained with dyes. After needle‑puncture‑induced disc degeneration, the stained hydrogels were injected into the damaged discs. Upon transverse sectioning of the harvested disc tissue and gentle irrigation with saline, the hydrogel was observed to adhere firmly within the disc space, demonstrating effective *in situ* retention (Supplementary Figure S4C).

### *In situ* injection of a PVA/Col/CS hydrogel for the treatment of IVDD in a rat model

**Figure 10:**
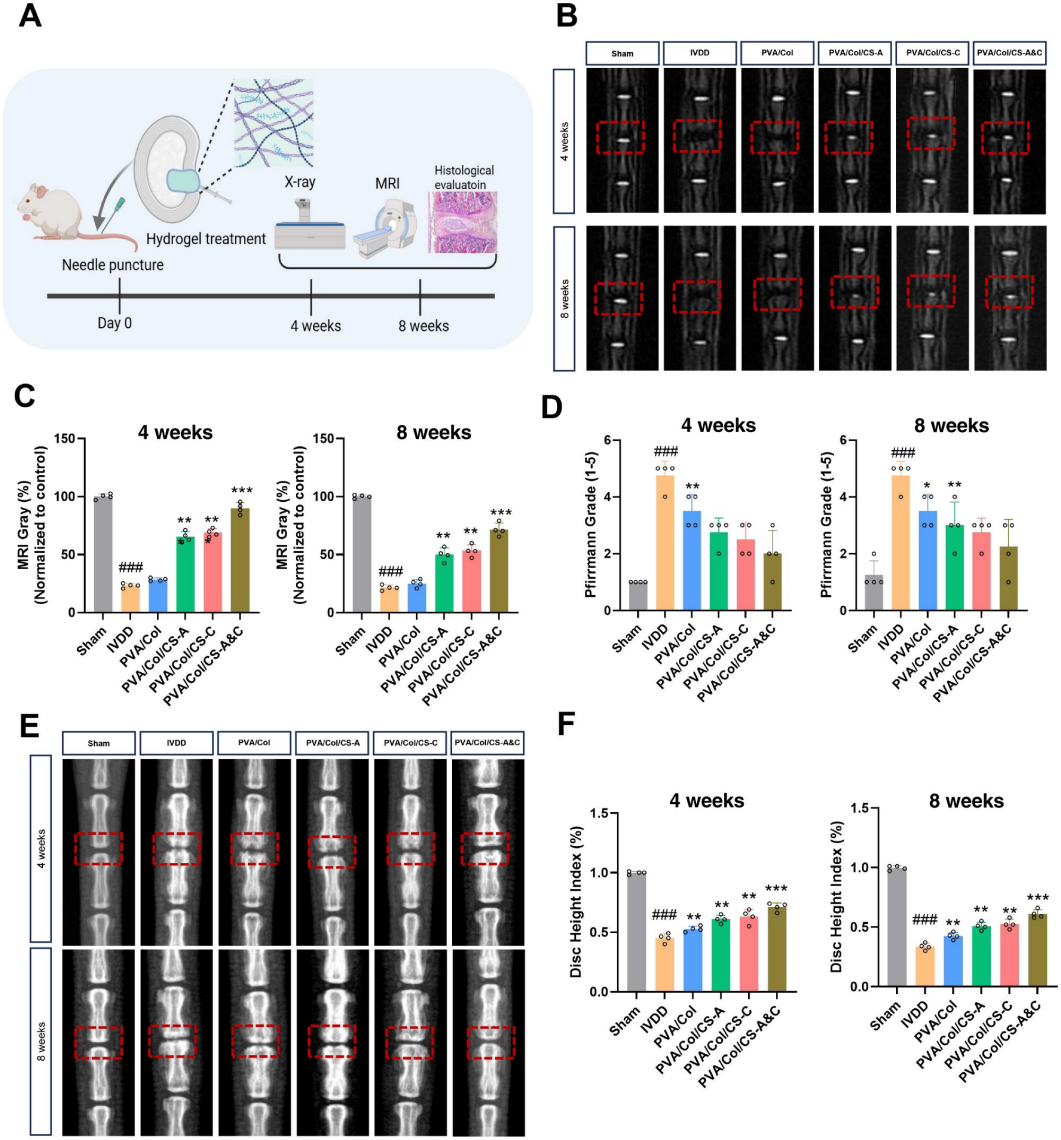
Implantation of the PVA/Col/CS hydrogel alleviates IVDD in a rat caudal model. (**A**) Schematic diagram of the needle puncture-induced IVDD model and subsequent hydrogel injection in rat caudal discs. Created in BioRender. Xin, W. (2026) https://BioRender.com/g8o9mok (Private repository link; will be made public upon manuscript acceptance.). (**B**) Representative T2-weighted MRI scans of the experimental groups. The red boxes highlight the target discs. (**C**) Quantitative analysis of the normalized signal intensity of the NP from the MRI scans in (**B**). (**D**) Pfirrmann grading scores for disc degeneration based on the MRI findings. (**E**) Representative X-ray images of rat tails at 4 and 8 weeks. The red boxes indicate the injected discs. (**F**) Quantitative analysis of the DHI from the X-ray images in (**E**). Data in (**C, D, F**) are presented as mean ± SD (*n* = 4). ^###^*P* < 0.001 vs. Sham group, *P < 0.05, **P < 0.01, ***P < 0.001 vs. IVDD group.

To assess the therapeutic efficacy of the CS-loaded hydrogel, we induced an IVDD model by needle puncture in rat caudal vertebrae and performed *in situ* injection into the intervertebral discs (Figure 10A).

No adverse systemic reactions were detected in any of the experimental animals during the entire study. Histopathological examination of major organs revealed no notable abnormalities, indicating favorable biocompatibility and a high safety profile of the hydrogel for *in vivo* application (Supplementary Figure S5A and B). Sagittal sections of the discs at the 4-week and 8-week time points post-injection indicated that the hydrogel groups, particularly the CS-A&C-loaded group, effectively preserved the disc structure (Supplementary Figure S6). T2-weighted MRI demonstrated a near-complete loss of signal intensity in the nucleus pulposus of the degeneration group, whereas the CS-loaded hydrogel groups exhibited significant signal recovery (Figure 10B). Specifically, the PVA/Col/CS group restored NP signal intensity to 85% at the 4-week time point and 72% at the 8-week mark, relative to the rats receiving the sham procedure (Figure 10C). The Pfirrmann grading system correspondingly confirmed the treatment efficacy of the PVA/Col/CS group at both 4 and 8 weeks (Figure 10D). X-ray imaging confirmed successful model induction, as evidenced by disc space narrowing, osteophyte formation and bone destruction at the vertebral margins in the degeneration group (Figure 10E). In contrast, the DHI of the PVA/Col/CS group recovered to 71% and 62% of the control value by Weeks 4 and 8, respectively (Figure 10F).

**Figure 11:**
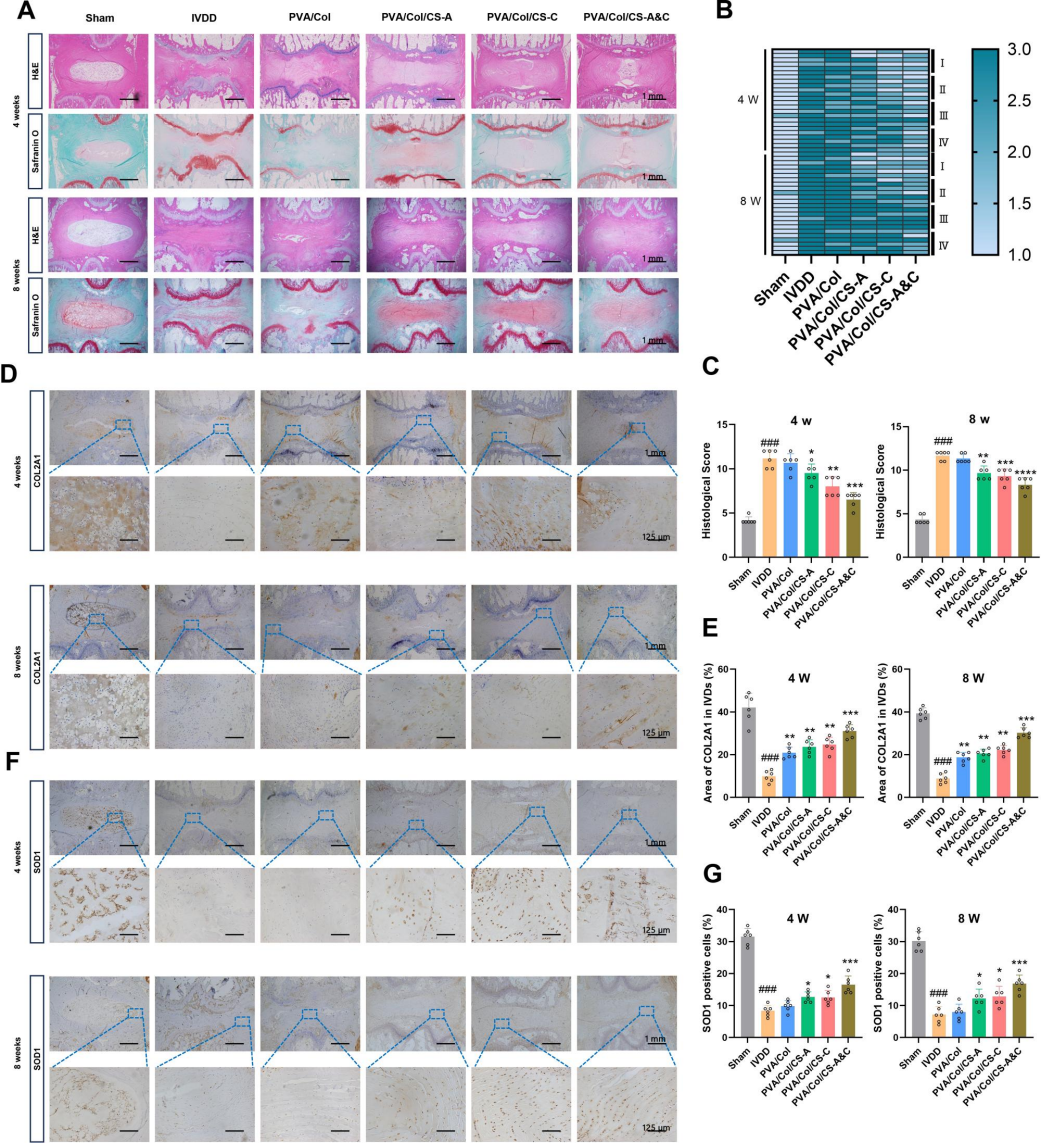
Histological and immunohistochemical evaluation confirms the therapeutic efficacy of the PVA/Col/CS hydrogel in promoting ECM synthesis and enhancing antioxidant capacity in a rat IVDD model. (**A**) Representative H&E and Safranin O-Fast Green (S-O) stained sagittal sections of intervertebral discs from different treatment groups at 4 and 8 weeks post-implantation. Scale bar: 1 mm. (**B**) Heatmap representing semi-quantitative histological scores based on the H&E and S-O staining. I–IV refers to 4 categories of degenerative changes based on Masuda scoring system [34]. (**C**) Quantitative analysis of the comprehensive histological scores presented in (**B**). (**D**) IHC staining for type II collagen (COL2A1) in disc tissues. Scale bars: 1 mm (overview) and 125 µm (magnified view). (**E**) Quantitative analysis of the percentage of COL2A1-positive area within the nucleus pulposus from (**D**). (**F**) IHC staining for superoxide dismutase 1 (SOD1) in disc tissues. Scale bars: 1 mm (overview) and 125 µm (magnified view). (**G**) Quantitative analysis of the percentage of SOD1-positive cells within the IVDs from (**F**). Data in (**C**, **E**, **G**) are presented as mean ± SD (*n* = 6). ^###^*P* < 0.001 vs. Sham group, *P < 0.05, **P < 0.01, ***P < 0.001, ****P < 0.0001 vs. IVDD group.

A histological evaluation of PVA/Col/CS hydrogel treatment was conducted. H&E staining revealed substantial ECM loss, blurred boundary between NP and AF, and pronounced narrowing of the intervertebral space in the degeneration cohort. While PVA/Col effectively maintained disc height, the CS-loaded groups demonstrated more preserved disc architecture (Figure 11A).

Safranin O-Fast Green staining indicated effective rescue of glycosaminoglycan (GAG) loss in the CS-loaded groups (Figure 11A). Histological scoring and corresponding quantitative analysis further corroborated these findings (Figure 11B and C).

Subsequently, we evaluated the expression of extracellular matrix-related markers as well as those involved in antioxidant defense in rat caudal intervertebral discs. Immunohistochemical and immunofluorescence analysis indicated a notable downregulation of COL2A1 in the degeneration cohort, confirming substantial ECM loss. The PVA/Col/CS hydrogel restored approximately 50% of COL2A1 expression relative to the control group at 8 wk (Figure 11D and E, Figure S7). Similarly, it restored 55% and 46% of SOD1 expression at 4 and 8 wk, respectively, demonstrating effective rescue of antioxidant capacity (Figure 11F and G).

## Discussion

Our study elucidates a dual-functional mechanism by which CS ameliorates IVDD, operating through the concurrent enhancement of endogenous antioxidant defenses and the promotion of anabolic matrix synthesis. Furthermore, we engineered a novel injectable hydrogel that integrates these biological insights with biomimetic design principles, demonstrating significant efficacy in a rat IVDD model. These findings collectively advance our understanding of CS biology in the disc and present a promising integrated therapeutic strategy. Initially, we confirmed the clinical relevance of CS depletion in human and rat degenerated discs, establishing a clear correlation between reduced CS levels and degenerative severity. This provided the foundational rationale for investigating CS beyond its conventional structural role, as a promising therapeutic candidate. We demonstrated that specific CS subtypes differentially promoted the synthesis and deposition of key matrix proteins in both NPCs and AFCs. Subsequent *in vitro* experiments robustly demonstrated that specific CS subtypes, particularly the combination of CS-A&C, could effectively counteract oxidative stress-a central driver of IVDD. The rescue of H_2_O_2_-induced ROS accumulation, alongside the upregulation of key antioxidant enzymes (SOD1, SOD2, CAT) and the master regulator Nrf2, underscores CS’s capacity to reinforce the disc cells’ intrinsic redox defense system. Importantly, this antioxidant effect was coupled with a potent pro-anabolic response, as evidenced by the restored expression of critical ECM markers (*Col1a1, Col2a1, Acan*) and proteins in both NPCs and NPCs. This dual action-scavenging damaging ROS while simultaneously rebuilding the ECM-addresses two fundamental pathological pillars of IVDD simultaneously, distinguishing our approach from singular-target strategies.

The high density of anionic charges on GAGs endows the nucleus pulposus with exceptional hydrophilicity and water-retention capacity, which is fundamental for resisting compressive axial loading. The homeostasis of GAG biosynthesis and catabolism is critically linked to disc integrity, and its dysregulation is a hallmark of degeneration [35]. Advancing the therapeutic promise of these native components, biomimetic GAG analogues have been engineered to promote ECM deposition while concurrently inhibiting aberrant neurite outgrowth [36]. Similarly, biomimetic proteoglycans have demonstrated superior hydration and enhanced resistance to enzymatic degradation compared to native aggrecan [37]. Among the disc’s GAGs, CS is predominant, constituting over 50% of the GAG dry weight in the human nucleus pulposus [38]. Its role, however, extends beyond structure. Notably, a GAG mimetic effectively lowers intracellular ROS, conserves mitochondrial membrane potential, and inhibits the cytochrome c-caspase-9/3 apoptotic cascade under oxidative stress [39]. Subsequent mechanistic studies attribute this cytoprotective effect specifically to CS and heparan sulfate, but not to heparin [39]. Consistent with its known bioactivity in promoting chondrogenic differentiation of mesenchymal stem cells (MSCs) [40], our findings reveal a novel and direct anabolic role of CS within native disc cells.

The central role of oxidative stress in driving the pathological cascade of IVDD is well-recognized [16, 17, 41]. Emerging evidence further highlights a complex interplay between oxidative stress and macrophage-mediated inflammation, where infiltrating macrophages amplify the inflammatory microenvironment and contribute to ECM degradation and neural ingrowth [42]. It acts as a key instigator that directly impairs cellular function by inhibiting anabolic activity, induces morphological alterations such as cell shrinkage and rounding, and critically disrupts mitochondrial integrity by compromising both their bioenergetic function and dynamic morphology [43, 44]. This multifactorial assault ultimately culminates in cell apoptosis, senescence and the catastrophic degradation of the ECM. Current research solidly positions the disruption of redox homeostasis not merely as a bystander but as a causative factor that perpetuates the degenerative cycle within the disc microenvironment [45]. In this study, we observed that CS treatment, especially CS-A&C, ameliorated oxidative stress-induced mitochondrial fragmentation, swelling and cristae disruption and restored membrane potential. Since mitochondria serve a dual role as the major generator of ROS and a central hub for cellular metabolism and apoptosis, their protection by CS provides a coherent explanation for the observed improvements in redox balance, cell viability and synthetic capacity [46].

To translate these cellular insights into a practical therapeutic intervention, we developed a multifunctional PVA/Col/CS hydrogel. This design strategically overcomes a major limitation in current disc regeneration strategies, which often target the nucleus or annulus in isolation. Our composite system provides: (1) Dynamic mechanical support via PVA to mimic load-bearing; (2) Bioactive cues via collagen to promote cell adhesion and function; and (3) Targeted delivery of CS to directly modulate the oxidative stress microenvironment and promote regeneration. PVA hydrogel is a water-based gel made from a synthetic polymer, known for its excellent mechanical strength, biocompatibility, non-toxicity and tunable properties [47–49]. While the inherent bio-inertia of PVA limits direct cellular interaction and bioactive signaling, collagen, as a primary native ECM component, offers intrinsic cell-adhesive motifs and bioactive cues that promote cellular attachment, proliferation and phenotypic expression [50–52]. The hydrogel’s inherent properties-injectability, self-healing, shear-thinning and controlled degradation-make it highly suitable for minimally invasive, *in situ* application. Most importantly, the loaded CS remained functionally active, as the hydrogel elicited significant therapeutic outcomes *in vivo*. Implantation of the PVA/Col/CS hydrogel into a rat caudal IVDD model effectively preserved disc structure, restored MRI T2 signal and disc height and improved histological scores. Crucially, IHC confirmed that the hydrogel successfully increased the expression of both COL2A1 (matrix synthesis) and SOD1 (antioxidant defense) within the treated discs, mirroring the dual effects observed *in vitro*. Based on these mechanistic and preclinical findings, we further discuss the clinical implications and potential translational directions of our CS-based hydrogel strategy. CS abundance may serve as a biomarker for IVDD severity. The dual antioxidant/anabolic effects of CS support its use in early-to-moderate degeneration. Our injectable hydrogel enables minimally invasive delivery and achieves integrated AF/NP repair. Future work should optimize CS sulfation, validate safety in large animal models and conduct randomized trials to compare CS hydrogel with current clinical treatments. Furthermore, systematic optimization of the CS‑A to CS‑C mixing ratio is warranted, as the equal‑ratio combination used in this study may not be optimal for all cell types or disease stages.

Despite these promising results, certain limitations of this study warrant consideration. First, while the rat caudal model is a well-established tool for preliminary IVDD studies, the pathophysiology and biomechanical environment differ from those in human lumbar discs. Future studies in larger animal models with axial loading will be essential for clinical translation. Second, the long-term stability of the hydrogel and the sustained release kinetics of CS *in vivo* require further optimization and investigation. Third, although we identified the integrin-PI3K-Akt pathway, the upstream signaling events triggered by specific CS sulfation motifs and their potential crosstalk with other pathways (e.g. TGF-β, MAPK) merit deeper exploration. For instance, TGF-β signaling could synergize with PI3K-Akt to promote matrix anabolism, while MAPK pathways might modulate inflammatory and stress responses. Fourth, the human disc tissue analysis correlating CS loss with Pfirrmann grade was based on a relatively small sample size (*n* = 15). Although a trend toward decreased CS positivity with increasing Pfirrmann grade (III, IV, V) was observed, statistical significance was not reached, likely due to the limited sample size. Future studies with larger cohorts are warranted to validate the quantitative relationship between CS abundance and degeneration severity.

## Conclusion

In conclusion, this work establishes CS as a potent dual-function agent that protects disc cells by reinforcing antioxidant defenses and promoting matrix synthesis via the integrin-PI3K-Akt-mitochondria axis. By embedding this bioactive molecule into a biomimetic, injectable hydrogel, we have created an integrated therapeutic system that addresses both the biological and structural facets of IVDD. This strategy moves beyond palliative care and singular-component regeneration, offering a holistic and translatable paradigm for functional disc restoration. Future work will focus on refining the hydrogel composition, elucidating detailed structure–activity relationships of CS sulfation patterns, and evaluating efficacy in more clinically relevant models.

## Supplementary Material

rbag134_Supplementary_Data

## Data Availability

The data that support the findings of this study are available from the corresponding author upon reasonable request.
