# Supplementary material for: Chondroitin sulfate reinforces mitochondrial redox homeostasis to enable integrated intervertebral disc regeneration
Source: Regen Biomater. 2026 Jun 15;13:rbag134. doi: 10.1093/rb/rbag134 (PMC13363253; doi:10.1093/rb/rbag134)
Supplement: rbag134_Supplementary_Data [file rbag134_supplementary_data.docx]

Supporting Information

Original article

Chondroitin Sulfate Reinforces Mitochondrial Redox Homeostasis to Enable Integrated Intervertebral Disc Regeneration


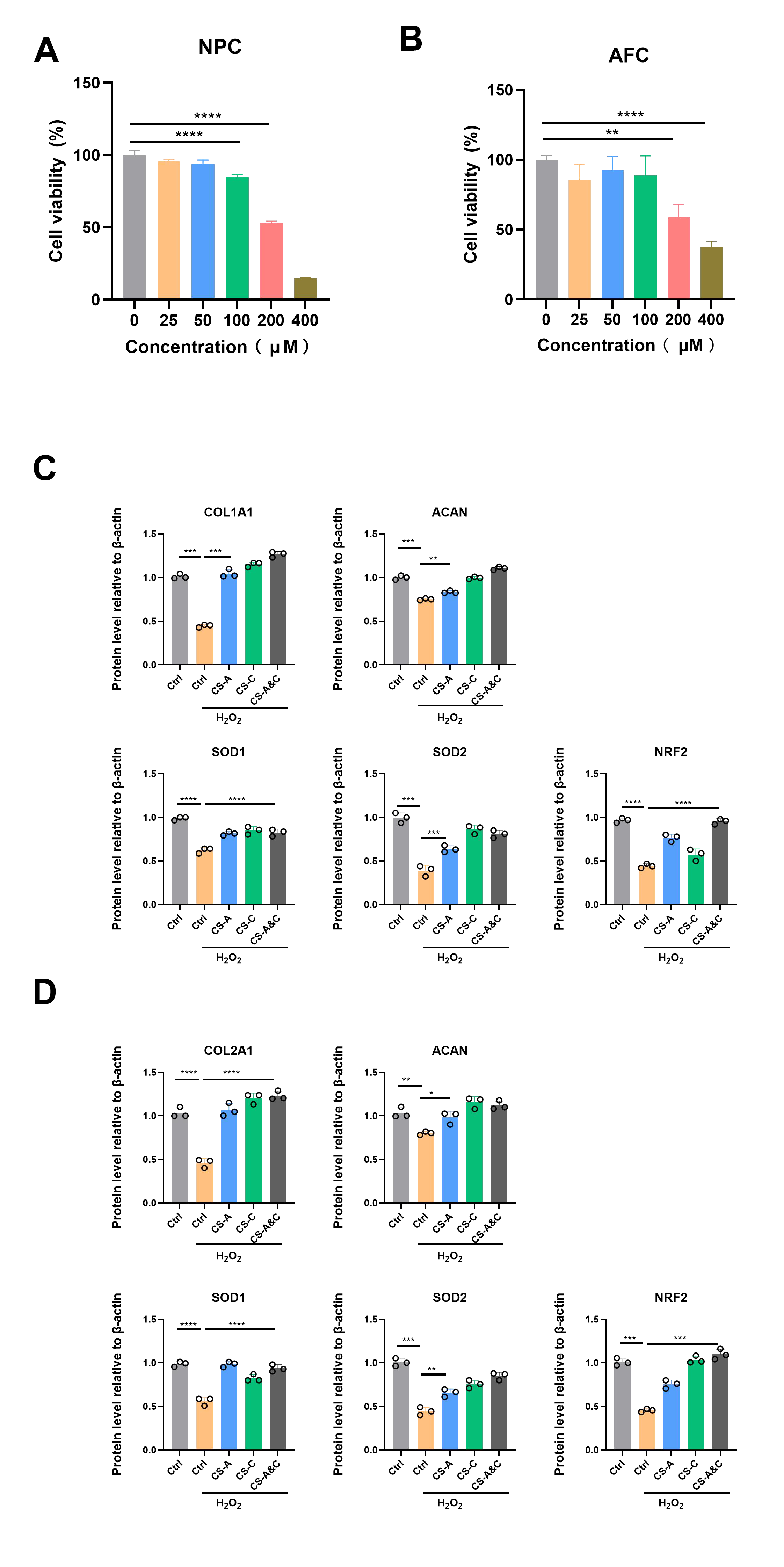


**Figure S1.** (A, B) Optimization of H₂O₂ concentration for inducing oxidative stress. Cell viability of rat NPCs (A) and AFCs (B) was assessed by CCK-8 assay after 2 h of treatment with the indicated concentrations of H₂O₂. Treatment with 200 µM H₂O₂ resulted in a reduction of cell viability by approximately 40-50% in both cell types. This concentration was selected for subsequent experiments as it induced significant oxidative stress while retaining a sufficient population of viable cells for intervention and molecular biology assays. (C, D) Quantitative analysis of matrix synthesis and antioxidant enzyme protein expression under oxidative stress. Bar graphs show the densitometric quantification of Western Blot bands from AFCs (C) and NPCs (D) treated with H₂O₂ (200 µM, 2 h) followed by intervention with different CS subtypes (CS-A, CS-C, or CS-A&C) for 3 d. Protein levels were normalized to β-actin. Data demonstrate the rescuing effect of CS subtypes on the expression of key proteins, including COL1A1, COL2A1, and antioxidant enzymes (SOD1, SOD2), which were suppressed by H₂O₂-induced oxidative stress. Data are presented as mean ± SD. **p < 0.01, ***p < 0.001, ****p < 0.0001.


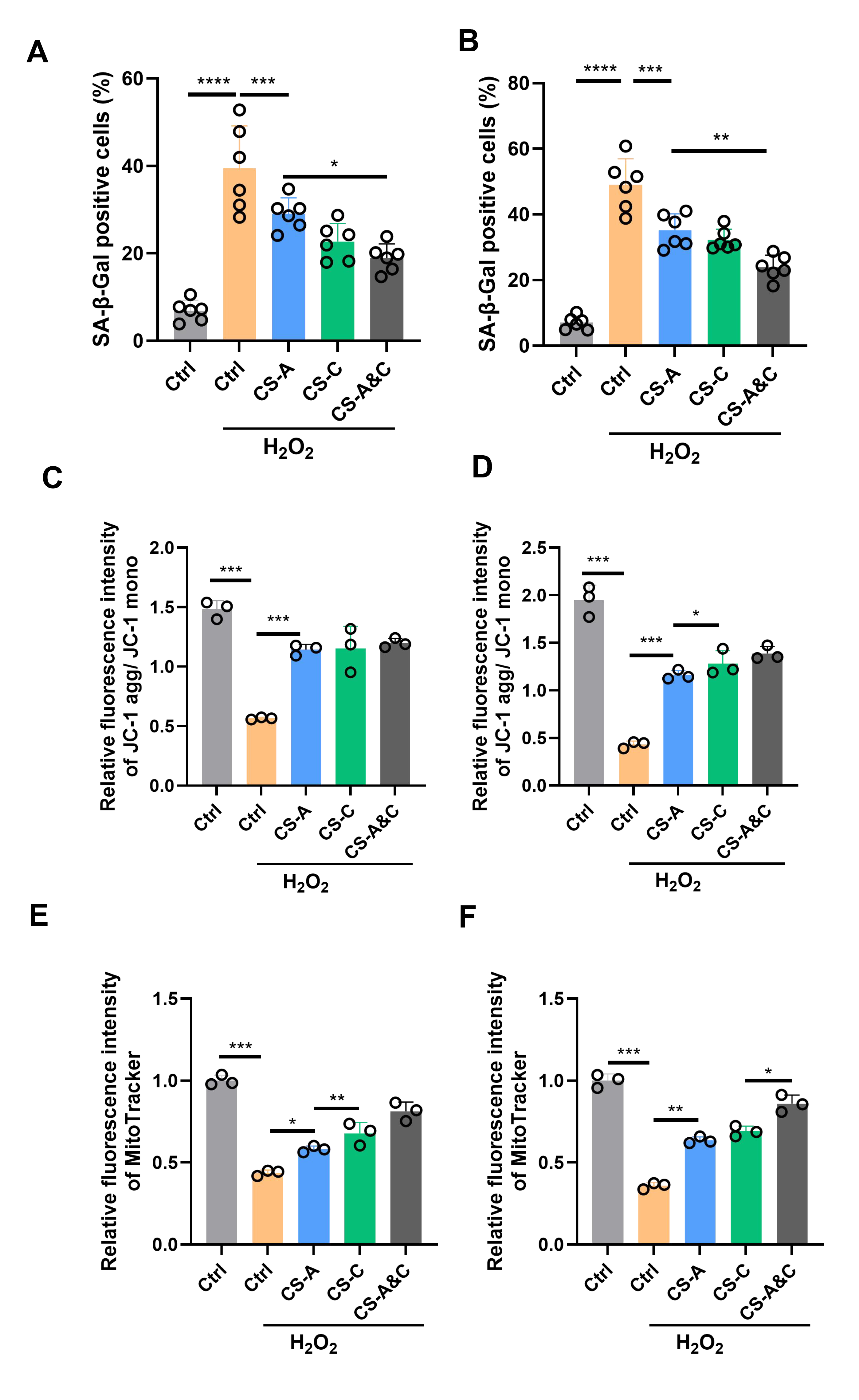


**Figure S2.** CS Attenuates Oxidative Stress-Induced Cellular Senescence and Mitochondrial Dysfunction in Disc Cells. (A) Quantitative analysis of senescence-associated β-galactosidase (SA-β-gal) positive AFCs under oxidative stress (200 µM H₂O₂, 2 h) and following treatment with different CS subtypes (CS-A, CS-C, CS-A&C). Data represent the percentage of SA-β-gal positive cells. (B) Quantitative analysis of SA-β-gal positive NPCs under the same experimental conditions as in (A). (C) Quantitative analysis of mitochondrial membrane potential in AFCs, assessed by the ratio of JC-1 aggregate (red, high potential) to monomer (green, low potential) fluorescence intensity, under oxidative stress and after CS intervention. (D) Quantitative analysis of mitochondrial membrane potential in NPCs, assessed by the JC-1 aggregate/monomer fluorescence intensity ratio under the same treatment conditions. (E) Quantitative analysis of MitoTracker Green fluorescence intensity in AFCs, reflecting mitochondrial content/mass, following H₂O₂-induced stress and CS treatment. (F) Quantitative analysis of MitoTracker Green fluorescence intensity in NPCs under the same experimental conditions. All quantitative data are presented as mean ± SD (n ≥ 3). Statistical significance: *p < 0.05, **p < 0.01, ***p < 0.001, ****p < 0.0001.


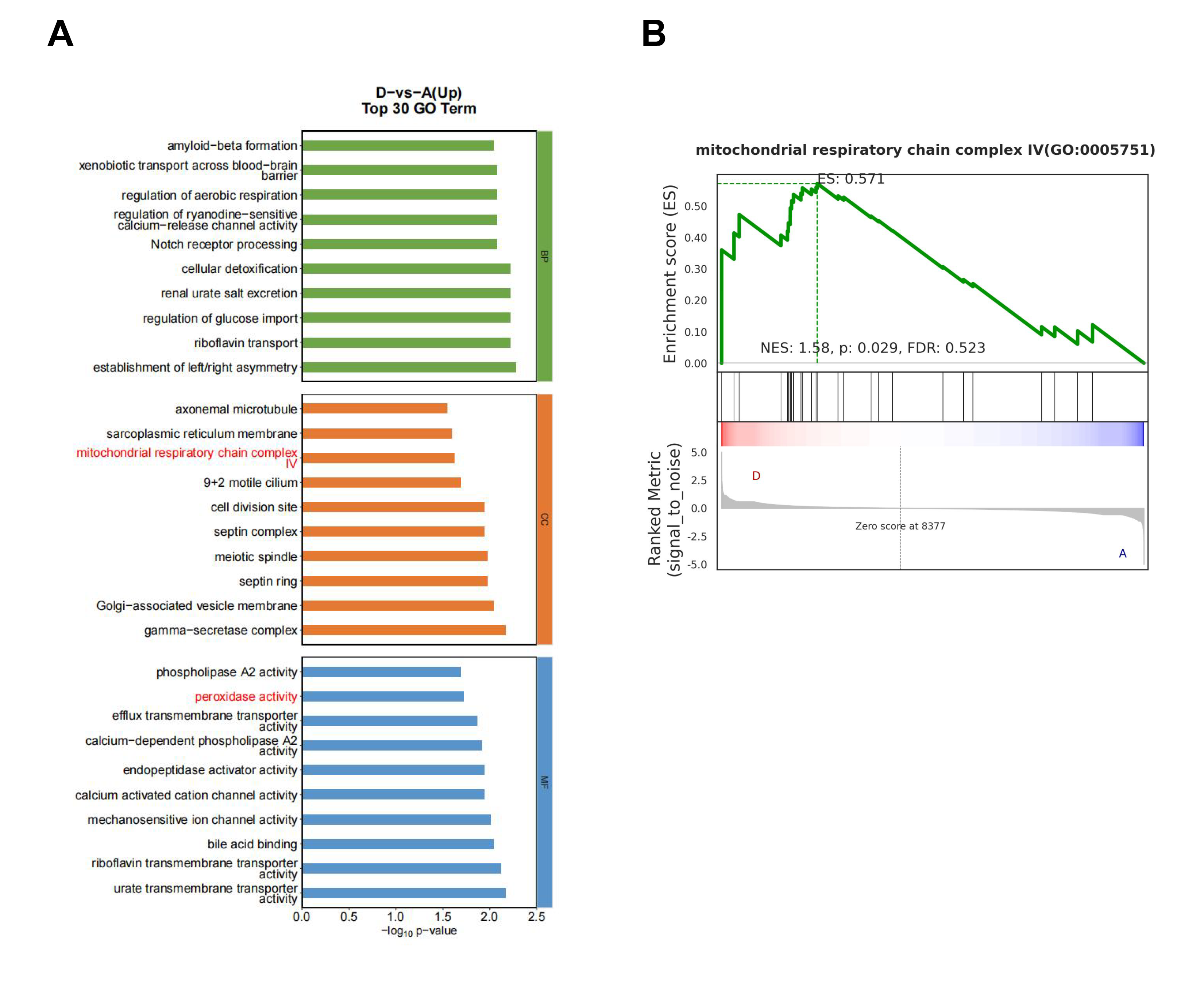


**Figure S3.** Transcriptomic Profiling Reveals CS Enhances Mitochondrial Oxidative Phosphorylation in AFCs. (A) Gene Ontology (GO) enrichment analysis of differentially expressed genes in AFCs treated with CS (combination CS-A&C) compared to untreated control. The bar chart displays the upregulation of pathways related to peroxidase activity and mitochondrial respiratory chain complex V (ATP synthase activity). (B) Gene Set Enrichment Analysis (GSEA) plot validating the significant upregulation of the gene set associated with mitochondrial respiratory chain complex V (ATP synthesis).


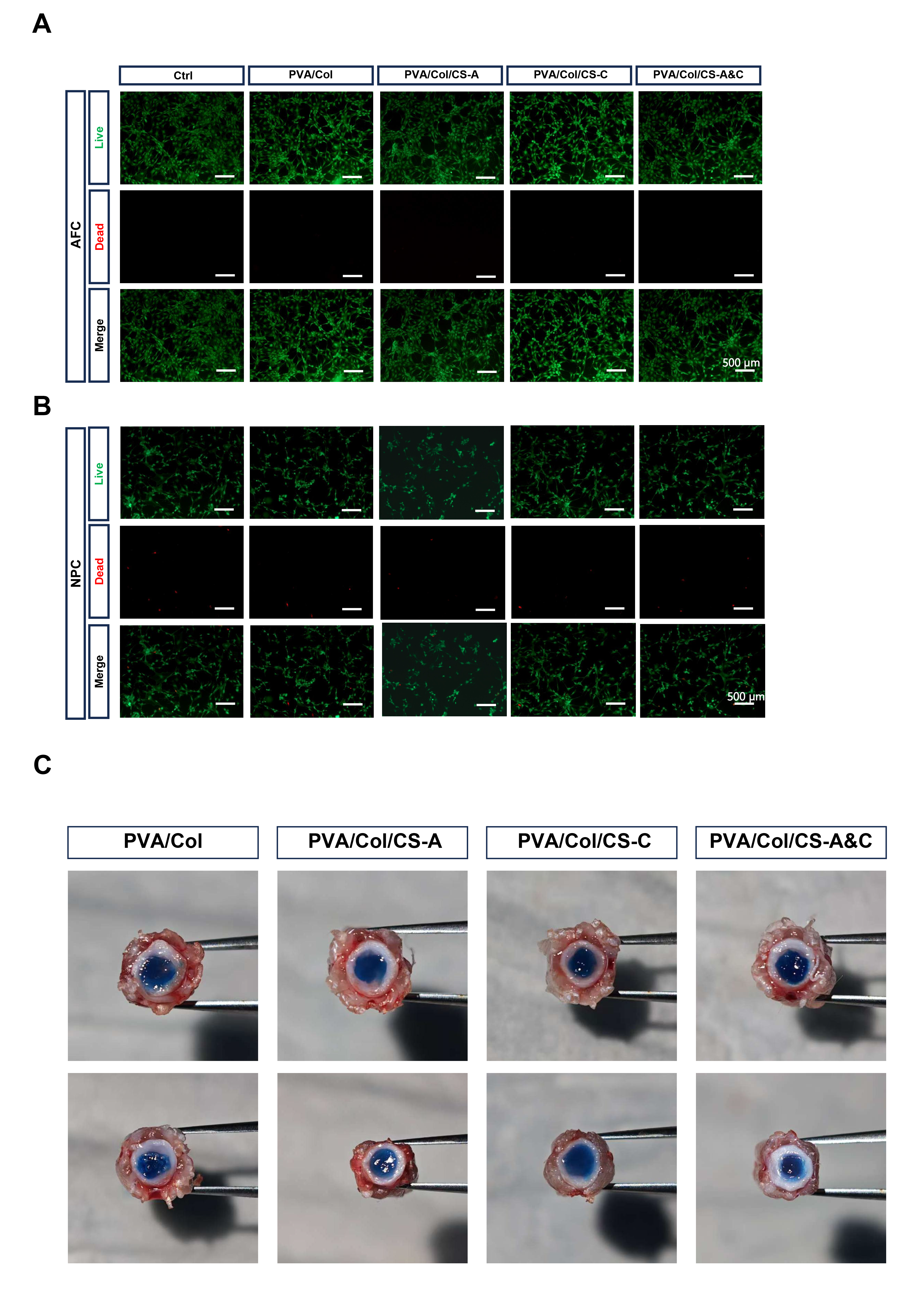


**Figure S4.** Biocompatibility and Adhesion of the PVA/Col/CS Hydrogel. (A-B) Live/dead staining of annulus fibrosus cells (AFCs) (A) and nucleus pulposus cells (NPCs) (B) cultured with extracts from different hydrogel formulations, demonstrating excellent biocompatibility across all groups. (C) Adhesion assessment of the hydrogels in situ. Following intradiscal injection, axial sections of the harvested discs were gently irrigated with saline, confirming strong and stable adhesion of the hydrogel within the disc space.

**Figure S5.** In Vivo Biocompatibility and Safety of the PVA/Col/CS Hydrogel. (A–B) Representative hematoxylin and eosin (H&E) staining of major organs (heart, liver, spleen, lung, kidney) harvested from rats at 4 wk (A) and 8 wk (B) post‑hydrogel implantation. No apparent histopathological abnormalities or signs of systemic toxicity were observed, confirming excellent in vivo biocompatibility and safety of the hydrogel system.


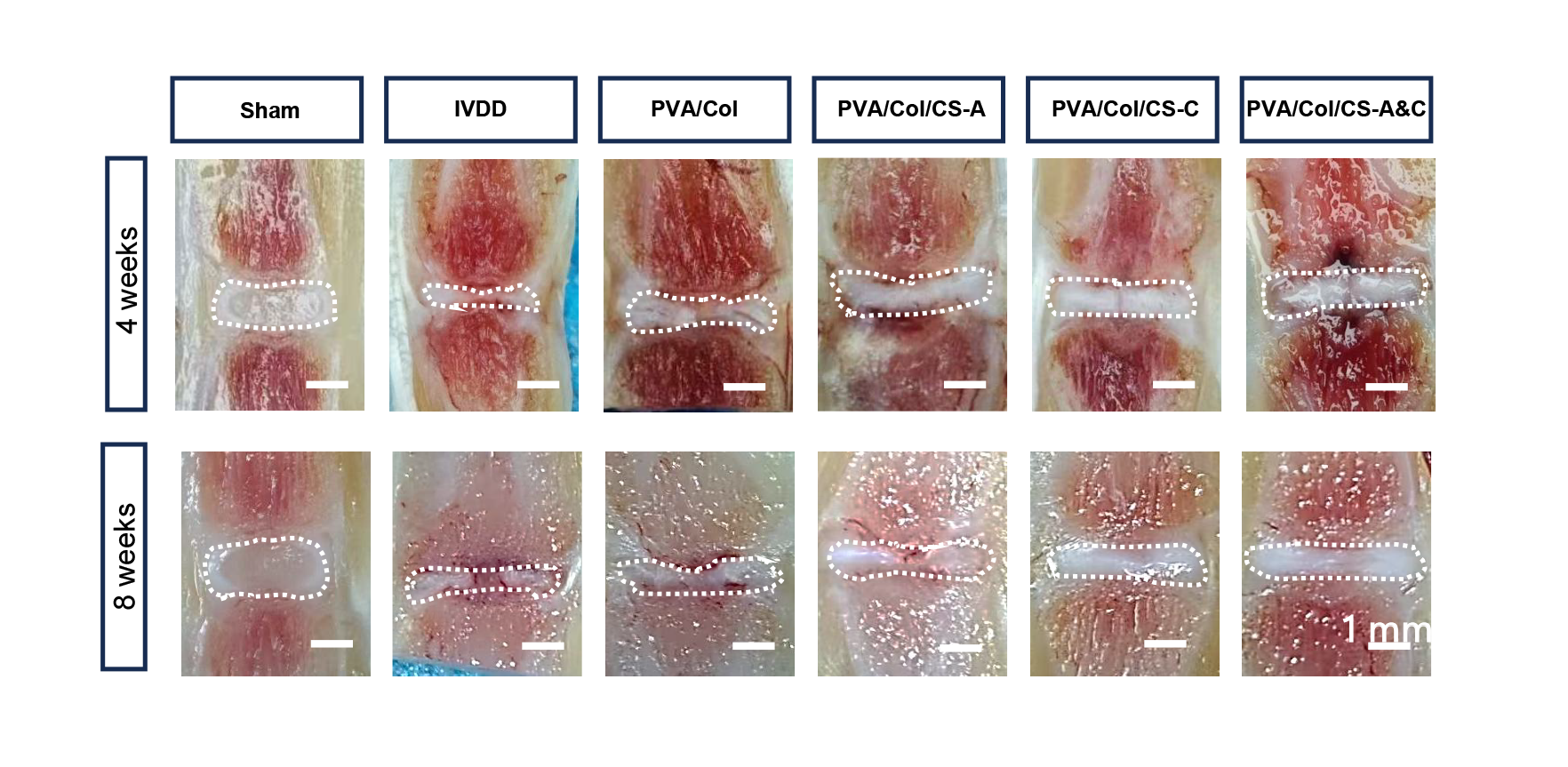


**Figure S6.** Macroscopic sagittal views of the caudal intervertebral discs at 4 and 8 wk post-operation. The CS-A&C hydrogel group shows improved disc structure preservation compared with the IVDD control. Scale bars: 1 mm.


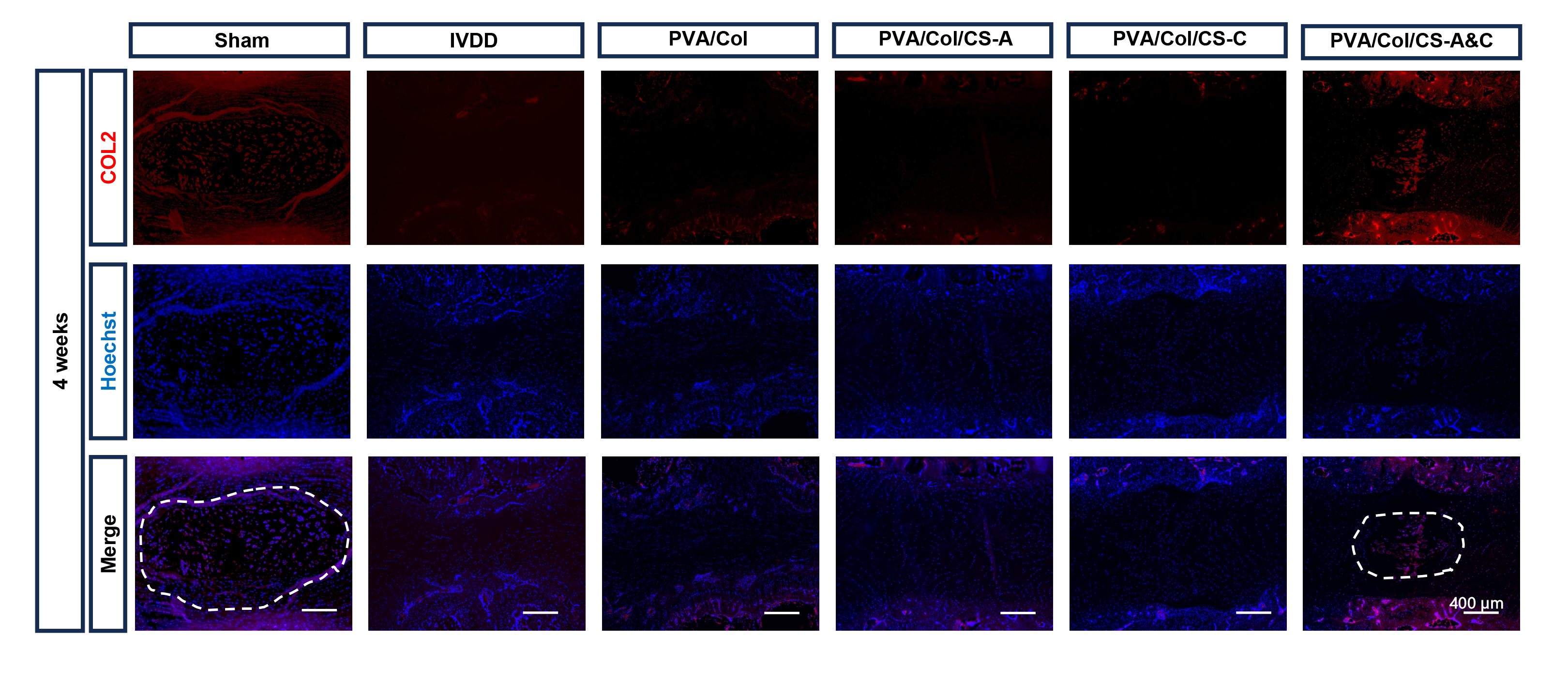


**Figure S7.** CS-A&C Hydrogel Promotes Extracellular Matrix Regeneration in IVD Tissues at 4 Wk Post-treatment. Representative immunofluorescence staining of COL2 in rat intervertebral disc tissues at 4 wk after intervention. Compared with the degeneration group, the CS-A&C group exhibited a marked upregulation of COL2 expression, particularly in the nucleus pulposus region, confirming the therapeutic efficacy of the composite hydrogel in promoting matrix anabolism.


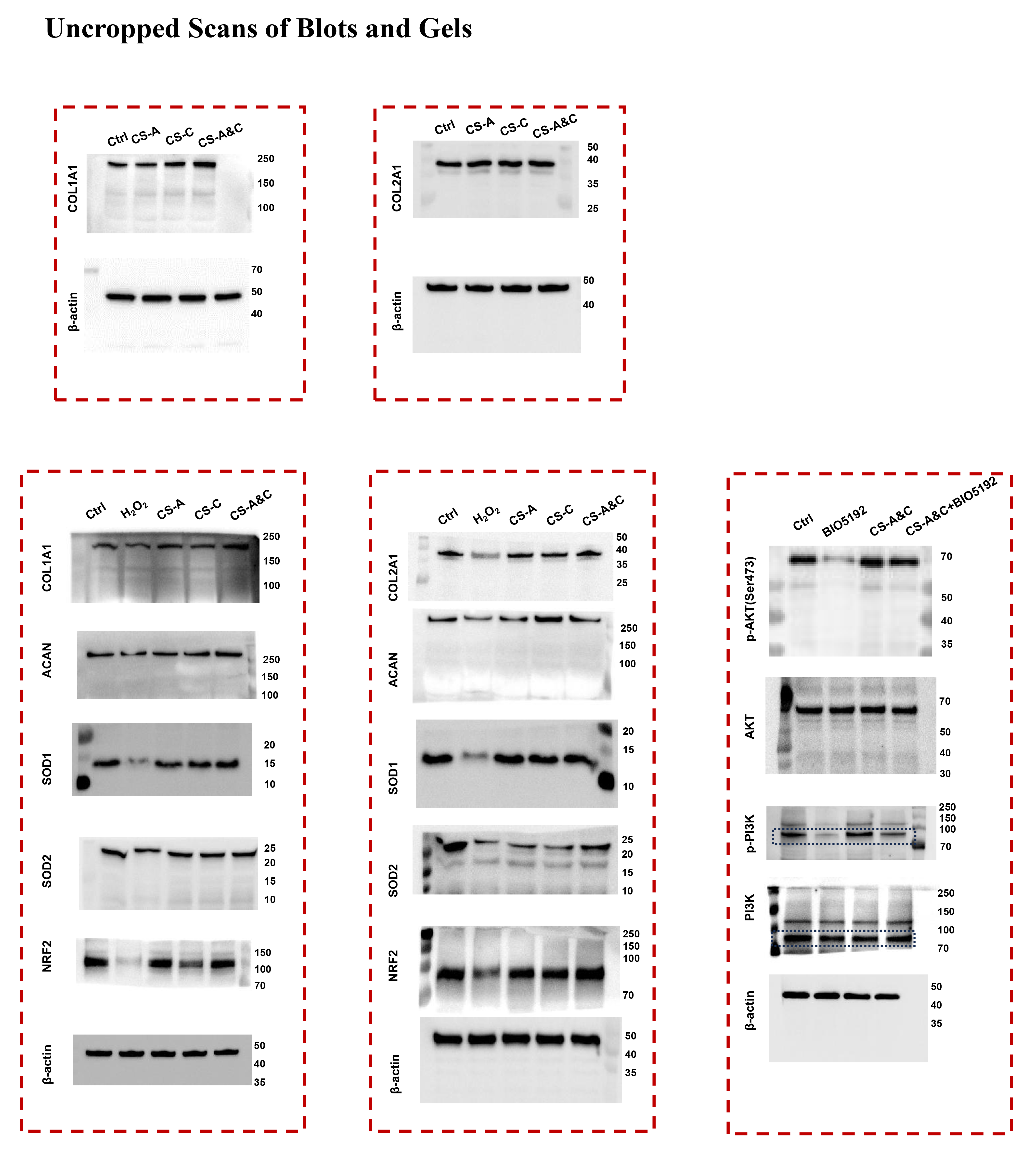


**Figure S8.** Uncropped raw Western blot membranes. Full‑membrane scans of all target proteins (COL1A1, COL2A1, ACAN, SOD1, SOD2, NRF2, p‑PI3K, PI3K, p‑AKT, AKT, β‑actin) are shown.

**Table S1.** Patients‘ information

| number | sex | age | disc level | Pfirrmann grade |
| --- | --- | --- | --- | --- |
| 1 | Femal | 84 | L4-5 | Ⅳ |
| 2 | Male | 39 | L5-S1 | Ⅲ |
| 3 | Femal | 55 | L4-5 | Ⅳ |
| 4 | Femal | 34 | L4-5 | Ⅴ |
| 5 | Male | 59 | L3-4 | Ⅲ |
| 6 | Femal | 39 | L5-S1 | Ⅲ |
| 7 | Femal | 27 | L2-3 | Ⅴ |
| 8 | Male | 45 | L5-S1 | Ⅲ |
| 9 | Femal | 42 | L5-S1 | Ⅳ |
| 10 | Male | 66 | L4-5 | Ⅲ |
| 11 | Femal | 36 | L3-4 | Ⅲ |
| 12 | Femal | 41 | L5-S1 | Ⅳ |
| 13 | Male | 31 | L4-S1 | Ⅳ |
| 14 | Femal | 69 | L4-S1 | Ⅲ |
| 15 | Femal | 42 | L4-5 | Ⅴ |

**Table S2.** Primer sequences for rat cells

| **Gene** | **Forward Primer sequence(5’-3’)** | **Reverse Primer sequence(5’-3’)** |
| --- | --- | --- |
| *Sod1* | CGTCATTCACTTCGAGCAGA | AAAATGAGGTCCTGCAGTGG |
| *Sod2* | GGCCAAGGGAGATGTTACAA | GCTTGATAGCCTCCAGCAAC |
| *Cat* | ACATGGTCTGGGACTTCTGG | CAAGTTTTTGATGCCCCTGGT |
| *HO-1* | GTAAATGCAGTGTTGGCCCC | ATGTGCCAGGCATCTCCTTC |
| *Nrf2* | GCTATTTTCCATTCCCGAGTTAC | ATTGCTGTCCATCTCTGTCAG |
| *Col2a1* | GGAGCAGCAAGAGCAAGGAGAAG | GGAGCCCTCAGTGGACAGTAGAC |
| *Col1a1* | TGTTGGTCCTGCTGGCAAGAATG | GTCACCTTGTTCGCCTGTCTCAC |
| *Acan* | CTGATCCACTGTCCAAGCACCATG | ATCCACGCCAGGCTCCACTC |
| *Gapdh* | CAAGTTCAACGGCACAG | CGCCAGTAGACTCCACGAC |
| *Gapdh* | GACATGCCGCCTGGAGAAAC | AGCCCAGGATGCCCTTTAGT |
